# CenH3 distribution reveals extended centromeres in the model beetle *Tribolium castaneum*

**DOI:** 10.1371/journal.pgen.1009115

**Published:** 2020-10-30

**Authors:** Tena Gržan, Evelin Despot-Slade, Nevenka Meštrović, Miroslav Plohl, Brankica Mravinac

**Affiliations:** Division of Molecular Biology, Ruđer Bošković Institute, Zagreb, Croatia; Fred Hutchinson Cancer Research Center, UNITED STATES

## Abstract

Centromeres are chromosomal domains essential for kinetochore assembly and correct chromosome segregation. Inconsistent in their underlying DNA sequences, centromeres are defined epigenetically by the presence of the centromere-specific histone H3 variant CenH3. Most of the analyzed eukaryotes have monocentric chromosomes in which CenH3 proteins deposit into a single, primary constriction visible at metaphase chromosomes. Contrary to monocentrics, evolutionary sporadic holocentric chromosomes lack a primary constriction and have kinetochore activity distributed along the entire chromosome length. In this work, we identified cCENH3 protein, the centromeric H3 histone of the coleopteran model beetle *Tribolium castaneum*. By ChIP-seq analysis we disclosed that cCENH3 chromatin assembles upon a repertoire of repetitive DNAs. cCENH3 *in situ* mapping revealed unusually elongated *T*. *castaneum* centromeres that comprise approximately 40% of the chromosome length. Being the longest insect regional centromeres evidenced so far, *T*. *castaneum* centromeres are characterized by metapolycentric structure composed of several individual cCENH3-containing domains. We suggest that the model beetle *T*. *castaneum* with its metapolycentromeres could represent an excellent model for further studies of non-canonical centromeres in insects.

## Author summary

Centromeres are specialized chromosome regions that mark a chromosomal “attaching site” from which the microtubules of the mitotic spindle pull the sister chromatids apart, ensuring accurate segregation of genetic material during cell division. Eukaryotic species studied to date mostly possess monocentric chromosomes with a functional centromere sited at a single locus, but there are also species with holocentric (polycentric) chromosomes whose centromeres stretch throughout the entire length of the chromosome. By investigating centromere-specific variant of the histone protein H3 and its associated centromeric DNA in the model beetle *Tribolium castaneum*, we discovered that *T*. *castaneum* centromeres comprise several functional domains merged into one remarkably extended region classified as a metapolycentromere. *T*. *castaneum* metapolycentromeres occupy approximately 40% of chromosome length, and emerge as the longest centromeres documented in insects to date. We believe that *T*. *castaneum*, as the representative species of the most numerous eukaryotic order Coleoptera could be a valuable model for future studies addressing atypical centromere structures.

## Introduction

Centromeres are chromosomal loci essential for chromosome pairing and accurate chromosome segregation during cell division. They represent a base upon which the kinetochore, a proteinaceous complex and the attachment site for spindle microtubules, is assembled. Despite centromere crucial role in chromosome inheritance and genome stability, organization of centromere domains varies among organisms [1,2]. From structural point of view, eukaryotic chromosomes are mainly monocentric, having functional centromere located within a single, cytologically distinct primary constriction. Regions of primary constrictions are often made up of highly repetitive DNA sequences such as satellite DNAs and mobile elements [3]. Contrary to monocentrics, holocentric chromosomes lack a primary constriction and have kinetochore activity distributed along almost the entire chromosome length. Holocentricity is less commonly found in eukaryotes studied so far, but it is assumed that it has arisen in different plant and animal lineages in multiple independent events [4]. In animals, holocentric chromosomes have been evidenced in nematodes [5] and arthropods [6] to date. A novel, possibly intermediate type of centromere structure has recently been identified in the plant genera *Pisum* [7] and *Lathyrus* [8], whose metapolycentric chromosomes comprise several centromere domains in a single, but remarkably elongated primary constriction.

Striking diversity of centromeric DNA sequences, not only between evolutionary distant species but also between closely related ones, led to a conclusion that there is no conserved DNA sequence that would be requisite or sufficient for centromere function. Neocentromeres, formed in ectopic sites upon anonymous sequences [9], as well as the new generation of the human artificial chromosomes that lack repetitive centromeric DNA [10], additionally challenge the idea of DNA sequence relevance. In the absence of a DNA cornerstone, the currently accepted definition of centromere identity is formulated epigenetically and relies on the presence of CenH3 proteins, centromere-specific histone H3 variants [11]. The human CENP-A was the first detected CenH3 protein [12], and afterwards its homologues have been identified in a wide range of eukaryotic organisms, from yeast to different animal and plant species [13]. Unlike the canonical histone H3, that is evolutionary well-conserved across eukaryotes, CenH3 proteins show considerable sequence variability at different taxonomic levels. The N-terminal tail is the most variable part of the amino acid sequence, making CenH3s often species-specific. On the other hand, the C-terminal part with a histone fold domain (HFD) is more conserved. In addition to the highly divergent N-terminal tail, certain changes in the HFD region are declared to be CenH3-specific, so they discriminate CenH3 variants from the canonical H3 and set bioinformatic criteria for identifying putative CenH3 *in silico* [14]. Although there have been evidenced species that lack CenH3 [6,15,16], CenH3 proteins still represent the most reliable markers of active centromeres. Nevertheless, great interspecies CenH3 varieties, corroborated by extremely divergent underlying DNA sequences, provoke the question of the centromere paradox where the function of the centromere is evolutionarily preserved while its DNA and protein constituents evolve rapidly [17]. The centromere drive hypothesis postulates that the rapid evolution of centromeric components could be triggered by centromeric sequences behaving as selfish genetic elements which drive non-Mendelian chromosome transmission during meiosis, thus stimulating the concurrent evolution of centromeric proteins to reconstitute fair segregation [17,18].

The red flour beetle *Tribolium castaneum* is an important world-wide pest of stored grain and grain products [19]. It is also the representative species of the Coleoptera, the most numerous and most diverse eukaryotic order with approximately 400000 species and 25% of all animal species described [20]. Due to easy rearing, plentiful offspring, relatively short life cycle, lots of mutants, and availability and proliferation of tools for its genetic analysis and manipulation, *T*. *castaneum* has been used in laboratory research for nearly 50 years, what makes it the most studied insect model system after *Drosophila* [21,22]. *T*. *castaneum* is also the first beetle whose genome has been sequenced [23]. In spite of that, 20% of the genome (44 Mb out of 204 Mb), primarily from the (peri)centromere regions, remained unmapped to genome assembly. The genome assembly with the contigs assembled into 10 chromosome groups was recently upgraded to the improved version Tcas5.2 [24], but the centromeric gaps are still unpatched. It was experimentally estimated that 17% of the *T*. *castaneum* genome is built of the highly abundant major satellite DNA TCAST that, according to cytological evidence, resides mainly in pericentromeres and centromeres of all *T*. *castaneum* chromosomes [25]. Since TCAST makes only 0.3% of the assembled genome [26], it can be assumed that this satellite is the major candidate for a centromere-eligible DNA sequence. As determined by reassociation kinetics, the repetitive DNA content in the *T*. *castaneum* genome is about 40% [27], and is mainly composed of tandemly repeated sequences and transposable elements residing for the most part within the chromosomal regions of low recombination [26]. Surprisingly, it was shown that at least 4% of the total genome sequence is comprised of nine satellite DNA families that are located principally in euchromatic portion of the *T*. *castaneum* genome [28]. Despite several elaborated studies on repetitive DNA content and distribution patterns in *T*. *castaneum*, its centromere regions as well as centromeric proteins have not been explored thus far.

In this work, we identified cCENH3 protein, a histone H3 centromeric variant of the beetle *T*. *castaneum*. By cCENH3 *in situ* mapping we provided evidence that cCENH3 occupies approximately 40% of *T*. *castaneum* chromosomes’ length, suggesting extraordinarily extended centromeres. By chromatin immunoprecipitation followed by high-throughput sequencing of cCENH3-associated DNA, we determined centromere-competent DNA sequences, among which the major satellite DNA dominates. This work represents the survey of centromeric regions in a species with unusually high content of a single repetitive DNA family, but also the pioneering experimental study of CenH3 proteins in the most species-rich eukaryotic order of Coleoptera in general.

## Results

### Identification of *Tribolium castaneum* cCENH3 coding sequence

In order to determine the CenH3 protein in *T*. *castaneum*, we performed an initial BLASTP search against the *Tribolium castaneum* OGS3 (Official Gene Set) database (http://beetlebase.org/blast/blast.html) using histone H3 protein sequence (NCBI Reference Sequence XP_966487.1) as a query. BLASTP search resulted in 13 reported hits (S1 Appendix). In addition to the five matches of canonical H3 to itself, BLASTP search revealed the gene ID TC012577 match that shared 42% identity (E-value 4e-19) with the H3 sequence. TC012577 has been annotated as a “histone H3-like protein” coding sequence according to the unreviewed computer-annotated UniProtKB/TrEMBL section. The remaining seven BLASTP matches showed significantly higher E-values from 0.8 to 8.8, covering the H3 sequence query only partially (S1 Appendix). From the 13 hits, TC012577 emanated as the most promising *T*. *castaneum* CenH3 candidate, and we named it cCENH3, as abbreviation for “castaneum CENH3”.

**Fig 1:**
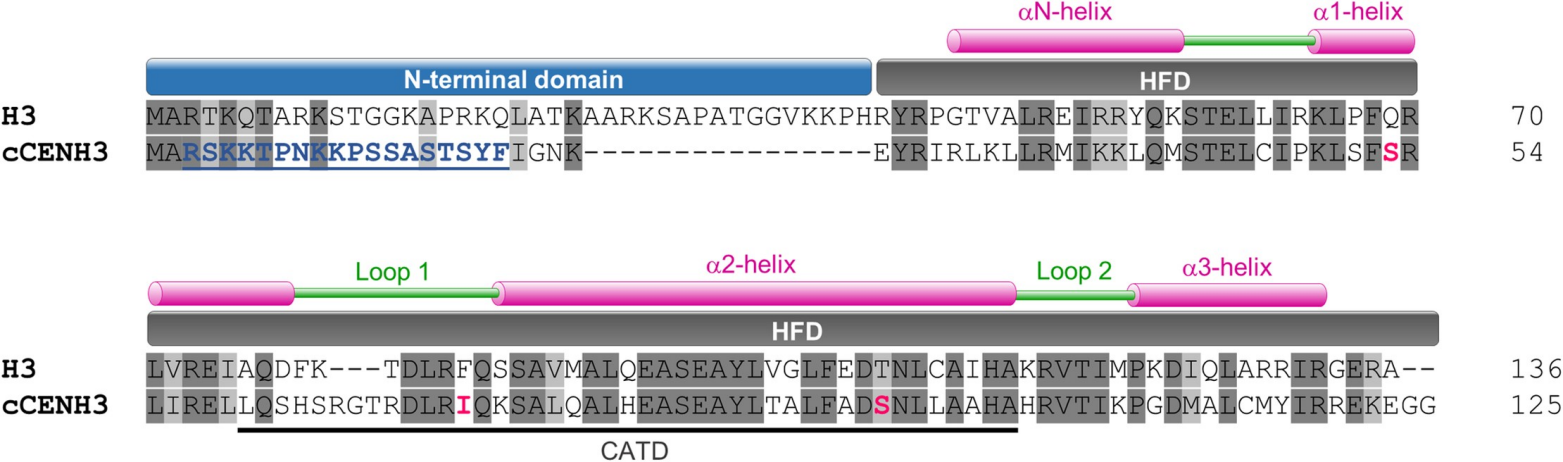
Protein sequence alignment of canonical histone H3 and its centromeric variant cCENH3 from *Tribolium castaneum*. The N-terminal domain and the histone fold domain (HFD) with its secondary structure are indicated above the alignment. The putative centromere-targeting domain (CATD) is marked under the cCENH3 sequence. The identical residues are highlighted in dark grey, while the similar residues are marked by light gray. The amino acid residues used for raising a peptide antibody against cCENH3 are highlighted and underlined in blue, and CenH3-characteristic amino acid changes are highlighted in red.

The protein sequence alignment of H3 and cCENH3 demonstrated that the major difference between the two proteins is the length and composition of N-terminal domains, which share only 18% identity in amino acid sequence (Fig 1). The histone fold domains (HFD) are more conserved showing 51% identity in pairwise comparison. In the HFD domain the cCENH3 protein shows the differences reputed to be characteristic for CenH3 variants: 1) a longer loop 1 region, and 2) absence of specific amino acids including glutamine, phenylalanine, and threonine at positions 69, 85, 108, respectively as compared to the canonical H3 (Fig 1).

We performed TBLASTN search using cCENH3 protein sequence to query the latest *T*. *castaneum* genome assembly Tcas5.2 [24] in order to examine whether multiple copies of the gene are present in the genome. The TBLASTN search mapped the *cCENH3* gene with 100% identity (E-value 1e-67) through entire length of the sequence only to one location at chromosome 9 (positions LG9:11651877–11652254) spanning a 378 bp long sequence. All the other matches, that showed remarkably lower significance (E-value >1.3e-12) covering the gene just partially (<62%), clearly suggest that the *T*. *castaneum* genome encodes a single copy *cCENH3* gene. By using *cCENH3* specific primers, we amplified and sequenced the *cCENH3* sequence from genomic DNAs isolated from three different *T*. *castaneum* strains, including GA2 that was used in *T*. *castaneum* genome sequencing project [23]. With the exception of a single synonymous substitution in one of the strains, the alignment of the sequenced PCR products showed no difference in DNA sequence between the three strains (S1 Fig), disclosing high conservation of the *CENH3* coding sequence between the strains.

**Fig 2:**
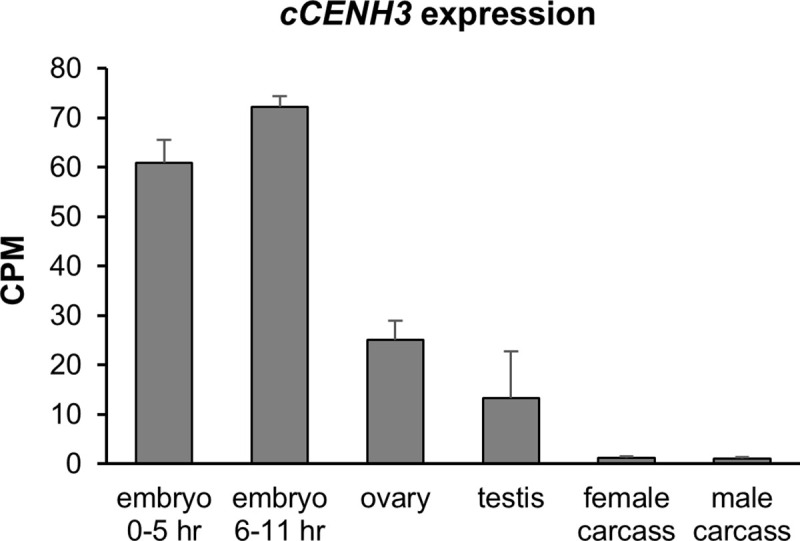
Expression of *cCENH3* gene. The expression profiles of cCENH3 gene in embryos (0–5 hr and 6–11 hr), germline (ovary, testis) and somatic tissues (female and male carcasses without gonads) were obtained from the original RNA-seq datasets from Khan *et al*. [29]. Transcript hits were normalized using CPM method. Error bars represent standard deviation calculated from two (testes) or three (all the other samples) biological replicates. Data on cCENH3 transcript hits are provided in S2 Appendix.

To confirm that the *cCENH3* gene is actively transcribed, reverse transcription was performed. RT-PCR reaction resulted in a unique band corresponding to ~380 bp fragments (S2 Fig). PCR products from amplification of cDNA template were cloned and sequenced. The *cCENH3* transcript sequence completely matched the coding sequence (S1 Fig), proving that the cCENH3 protein is encoded by a 378 bp long gene with no introns. We examined the expression of *cCENH3* in *T*. *castaneum* embryo, germline and somatic tissues by analyzing publicly available transcriptome datasets from Khan *et al*. [29]. We found that the *cCENH3* expression was the highest during embryonic development, and the expression profiles of the two embryo stages (0–5 hr and 6–11 hr) were similar (Fig 2, S2 Appendix). Compared to embryos, the ovaries and testes showed 2.4–5.4x lower expression, while the expression is drastically decreased in adult female (without ovaries) and male (without testes) carcasses (Fig 2, S2 Appendix). Such expression pattern is concordant with the significantly higher cell division activity in embryogenesis and in germline compared to somatic tissues.

### Chromosomal localization of the cCENH3 protein

We further produced an antibody specific for the N-terminal domain of cCENH3 (Fig 1). Western blot on the whole protein extract from *T*. *castaneum* revealed that the rabbit-raised antibody recognizes a protein of around 15 kDa (S3 Fig), consistent with cCENH3 predicted molecular weight of 14.23 kDa.

**Fig 3:**
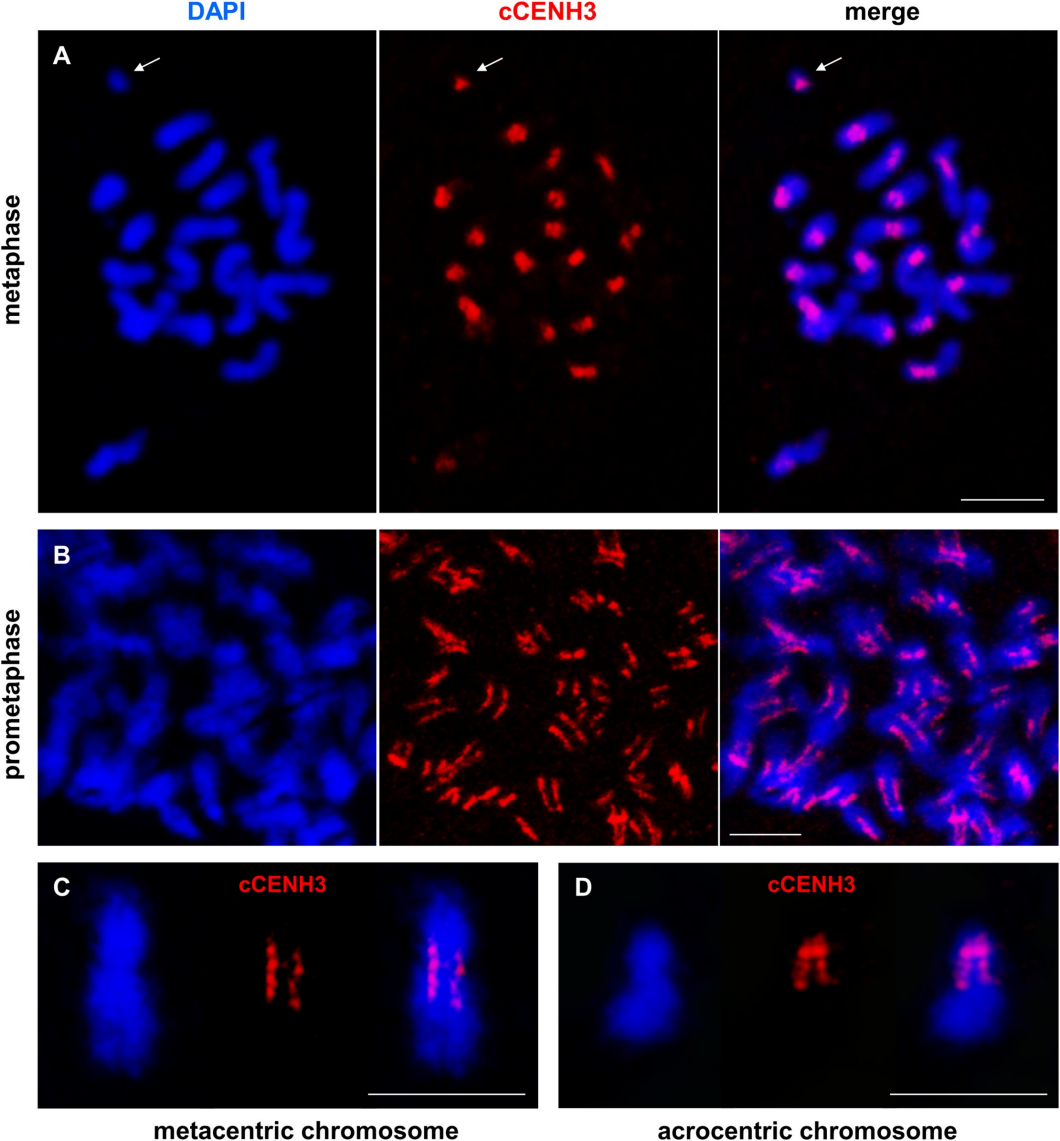
Localization of cCENH3 protein on *Tribolium castaneum* chromosomes. The position of cCENH3 protein (shown in red) was determined on *T*. *castaneum* chromosomes (shown in blue) by immunodetection using cCENH3 antibody. (A) On metaphase chromosomes cCENH3 localizes to the primary constrictions of all chromosomes including the minute y_p_ chromosome (marked by an arrow). (B) Prometaphase chromosomes show extended cCENH3-containing regions with bead-like signal patterns composed of multiple cCENH3-containing domains. Enlarged view of metapolycentromeres with cCENH3-containing domains separated by cCENH3-lacking segments is shown at metacentic (C) and acrocentric (D) chromosome. The chromosomes are counterstained with DAPI. Scale bars = 5 μm.

Using the cCENH3 antibody, we conducted immunofluorescence (IF) experiments on *T*. *castaneum* chromosome spreads. *T*. *castaneum* (2n = 20) has a karyotype based on 18 autosomes and a sex chromosome pair, which is composed of two relatively large X chromosome in females, while males have a Xy_p_ parachute-like association based on the X and a minute y_p_ chromosome [30]. Immunodetection of cCENH3 on metaphase spreads revealed paired signals at primary constriction of all chromosomes (Fig 3A), thus confirming that cCENH3 is indeed a centromeric histone. *T*. *castaneum* chromosomes differ in their size and form, and therefore the position of cCENH3 signals varies between chromosomes reflecting their metacentric, submetacentric or acrocentric architecture.

**Fig 4:**
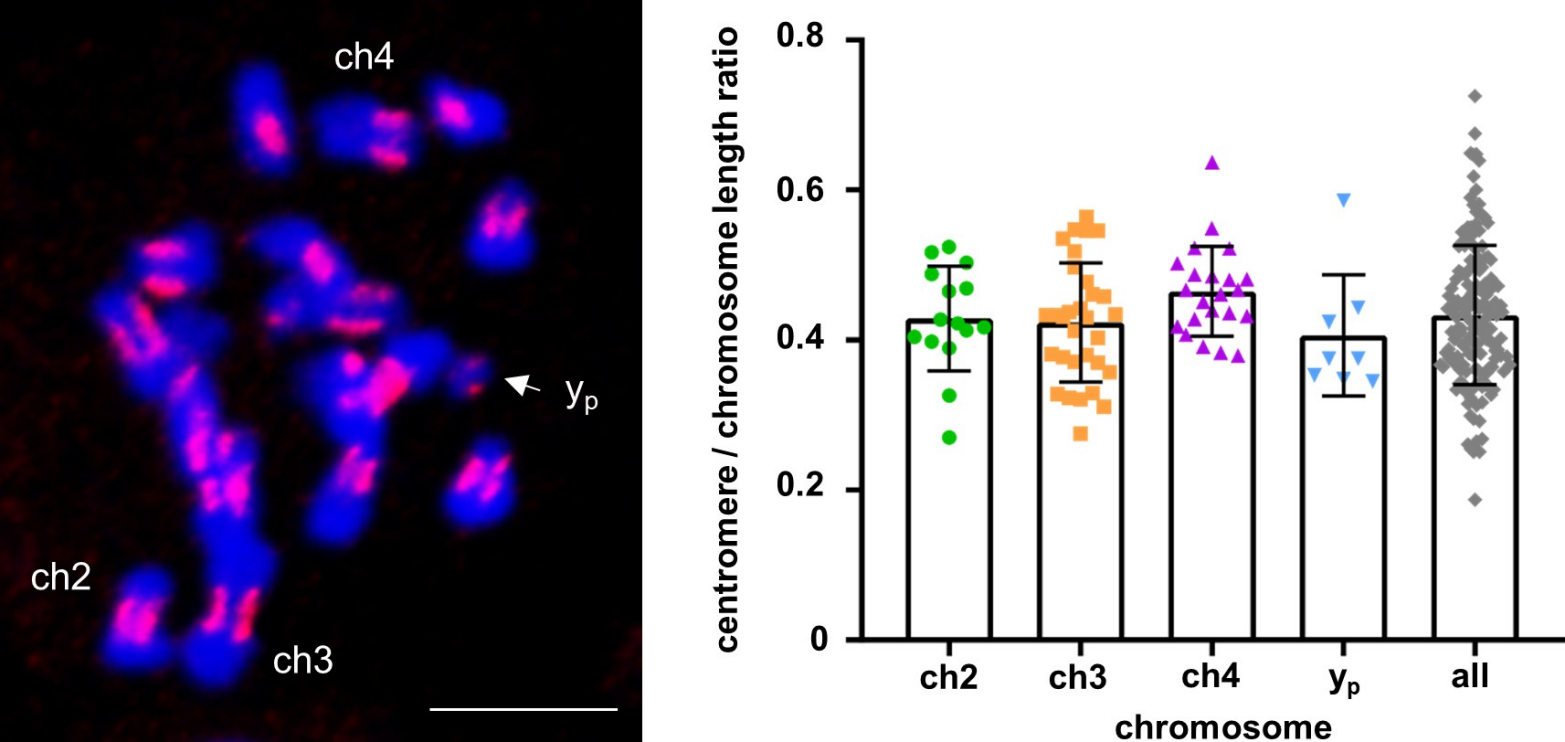
*Tribolium castaneum* relative centromere length estimation. Centromere length relative to chromosome length was calculated from 240 measured chromosomes. The left panel shows a representative chromosome spread with DAPI-stained chromosomes (shown in blue) with red-stained cCENH3 regions. Centromere length was defined as the distance between the two outermost cCENH3-containing domains at each chromosome. The scatter plot displays values for all measured chromosomes, and for the individual chromosomes ch2, ch3, ch4 and y_p_ that can be distinguished in the chromosome complement (marked in the left panel, scale bar = 5 μm). Error bars represent standard deviations of the underlying measurement data supplied in the S3 Appendix.

Interestingly, cCENH3 distribution at prometaphase chromosomes did not demonstrate a typical centromeric dot-like signal. Instead, elongated cCENH3 signals disclosed extended centromeric regions (Fig 3B), which are best apparent at the metacentric (Fig 3C) and acrocentric (Fig 3D) chromosomes. Moreover, prometaphase chromosomes revealed the bead-like cCENH3 signals composed of several, in most instances four, individual cCENH3-containing domains (Fig 3B–3D). This form of “metapolycentric” signal has been evidenced irrespectively to the centromere position on the chromosome (Fig 3C and 3D). To estimate the relative size of the extended *T*. *castaneum* centromeres, the distances between the two outermost cCENH3-containing domains were measured. Based on measurement of 240 prometaphase chromosomes, the centromere regions average 43.5% of the chromosome length (Fig 4, S3 Appendix). Due to relative small size (1–5 μm in the most condensed form), *T*. *castaneum* chromosomes cannot be easily distinguished [30]. However, some of them can be recognized as the longest metacentric autosome ch3 (LG3), the second largest metacentric chromosome ch2 (LG2), the largest acrocentric chromosome 4 (LG4), and the smallest chromosome y_p_ (Fig 4). According to the measurements performed on these four chromosomes, the cCENH3 signals occupy 40.6–46.5% of their length (Fig 4, S1 Table, S3 Appendix). Although the exact calculation is not possible due to different level of chromatin condensation in euchromatic and heterochromatic regions, we estimate that the longest centromere of approximate 15.8 Mb belongs to the longest chromosome ch3 (LG3), while the minute y_p_ chromosome has the smallest centromere of about 2.3 Mb. It has to be stressed that in contrast to the other *T*. *castaneum* chromosomes that exhibit bead-like cCENH3 distribution, the y_p_ shows a single, dot-like cCENH3 signal (Fig 4). Notwithstanding the bead-like or dot-like patterns, the cCENH3 signals are always located at the poleward surfaces of the primary constrictions (Figs 3 and 4), as expected for a centromeric histone H3 variant.

### Detection of cCENH3-associated DNA sequences

In order to elucidate DNA sequences associated with cCENH3 at *T*. *castaneum* centromeres, we applied a native chromatin immunoprecipitation (ChIP) approach using the antibody directed against cCENH3. DNA extracted from cCENH3-immunoprecipitated chromatin was fluorophore-labeled and fluorescence *in situ* hybridization (FISH) experiment was performed. Although the intensity and the size of FISH signals were not uniform, the cCENH3-ChIPped DNA probe hybridized to centromeric regions of all *T*. *castaneum* chromosomes (S4 Fig). The cCENH3-ChIPped DNA signals cover on average 45.8% of the chromosome length (S4 Appendix), which is in agreement with the estimated average size of cCENH3 regions (Fig 4). The position of the cCENH3-ChIPped DNA signals corresponds to the acrocentric and submetacentic morphology previously presumed for the majority of *T*. *castaneum* chromosomes [30]. This result provided cytological evidence for centromere specificity of cCENH3-ChIPped DNA material and suggested a large amount of repetitive DNA in *T*. *castaneum* centromeres.

**Fig 5:**
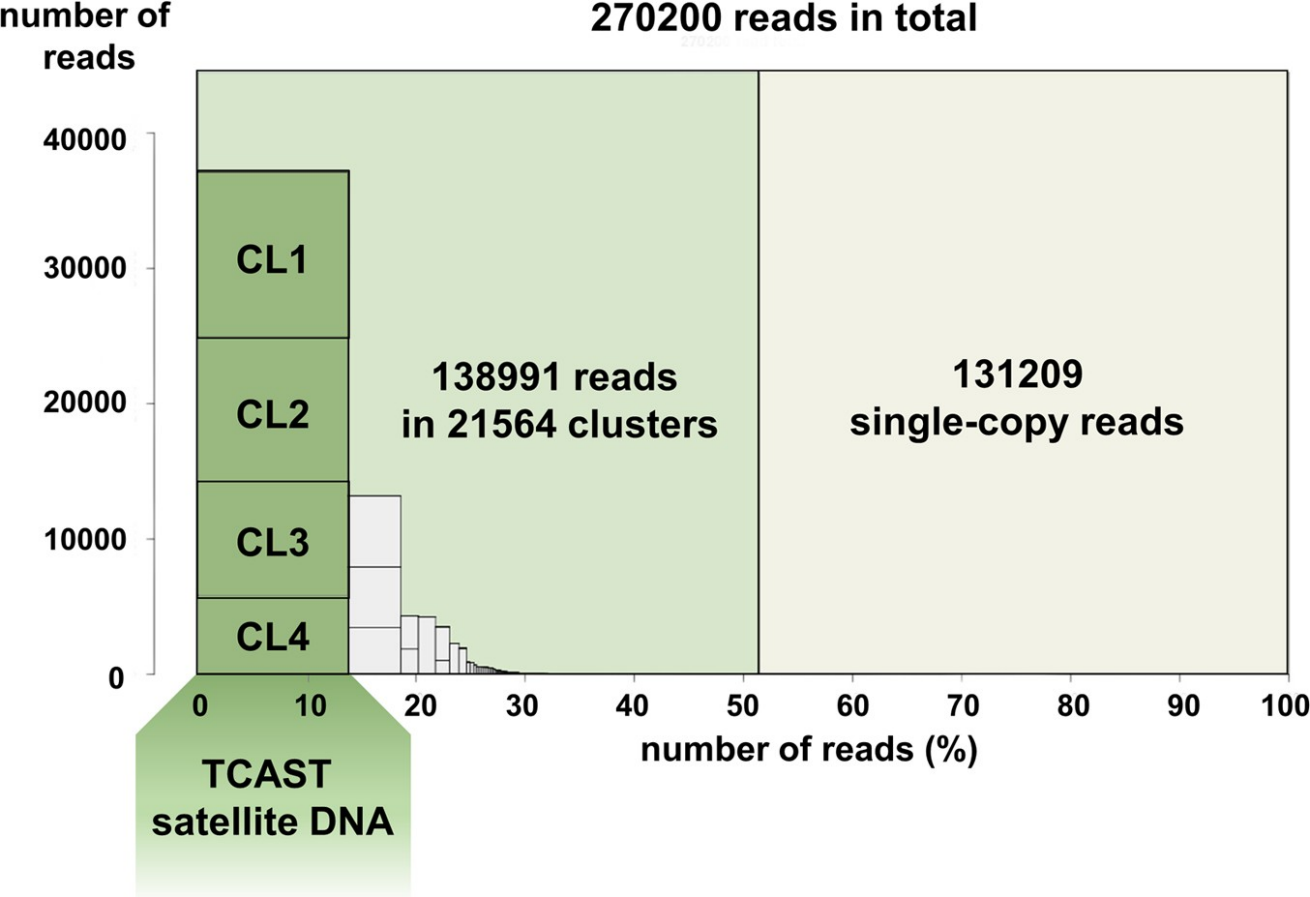
Summary of the *Tribolium castaneum* genome clustering. 270220 Illumina reads corresponding to 0.2x genome coverage were analyzed using RepeatExplorer2 pipeline [31]. 138991 reads were assorted into 21564 clusters representing repetitive fraction of genomic DNA, while 131209 reads were classified as single-copy reads. The four most highly-repetitive clusters (CL1-CL4, marked by dark green columns) belong to the supercluster of *T*. *castaneum* major satellite DNA TCAST, and cumulatively comprise 13.8% of the genome.

Next, to investigate the identity of cCENH3-ChIPped DNA sequences, we performed chromatin immunoprecipitation followed by sequencing (ChIP-seq). Because of the assumed high DNA repetitiveness of centromeric regions, in the ChIP-seq data analysis we applied the strategy introduced by Neumann *et al*. [7]. In this approach, the repetitive DNA content of the genome is first classified into clusters according to the graph-based repeat clustering analysis [31], and the output of the annotated clusters serves as a reference for similarity-based mapping of ChIP-Seq reads. The advantage of this approach is that it does not depend on a genome assembly, and is optimized for analyzing next-generation sequence reads, as the algorithm uses short sequences randomly sampled from the low pass genome sequencing data. The workflow of the cCENH3-ChIP-seq experiments and data analysis is presented in S5 Fig. The *T*. *castaneum* genome has been assembled into ten chromosome/linkage groups, but the current Tcas5.2 assembly lacks 20% of the genome sequence attributed to the heterochromatic (peri)centromeric regions [23,24,26], so it could not serve as a reliable repeat database reference. Therefore, we re-sequenced the *T*. *castaneum* genomic DNA using the Illumina HiSeq platform. From the low pass genome sequencing data and graph-based sequence clustering obtained by RepeatExplorer2 analysis, we generated the repeat database directly from the unassembled Illumina WGS reads. The repeat reference database was constructed from 270200 randomly selected, 151 nt long WGS reads that ensured 0.2x genome coverage, determined as optimal (as explained in the Materials and Methods section). Based on the RepeatExplorer2 analysis, 51.44% of the WGS reads were classified into 21564 clusters, while the rest (48.56%) represents ungrouped, singleton reads (Fig 5). As the clusters’ order reflects their genome abundance, the first 1000 clusters were used for subsequent similarity-based mapping of ChIP-seq reads, while the rest were omitted from the analysis due to their low representation. For ChIP-Seq Mapper analysis, one million reads were randomly selected for the cCENH3-ChIPped sample together with one million reads randomly selected for the input sample (DNA isolated from an aliquot of native chromatin prior to ChIP). The ratio of ChIP to input read hits was calculated for the 1000 WGS reference clusters. The ChIP-Seq Mapper analysis set the mean ratio between ChIP and input hits to 2 (S6A Fig), propounding it as a ChIP enrichment threshold that 37 out of 1000 analyzed clusters exceeded (S6B Fig). However, as the enrichment threshold was set relatively low, when judging the centromere competence of a certain cluster, the number of supporting ChIP hits has to be considered. For that reason, we proceeded to further analysis only with the >2-fold enriched clusters that were supported by at least 0.01% of ChIP hits (i.e. 100 ChIP hits) (Table 1). We also included into analyses additional seven clusters showing ChIP enrichment >1, but being represented with >10000 ChIP hits (>1% of the analyzed reads). The clusters that met mentioned criteria (Table 1) were mapped to the *T*. *castaneum* Tcas5.2 genome assembly. Strikingly, we mapped them largely outside the assembled regions, in unplaced scaffolds and singletons, or in the arrays adjoining the unassembled regions. The clusters listed in the Table 1 were also BLAST-searched against Repbase and GenBank databases to reveal the possible identity or similarity with annotated sequences. According to similarity-search analysis, the clusters enriched in cCENH3-ChIP-seq data can be classified into four groups (Table 1): tandemly repeated sequences or satellite DNAs, transposable elements, rDNA-like sequences, and anonymous sequences.

**Table 1:** Characterization of cCENH3-associated repetitive clusters.

Criteria	Cluster^1^	ChIP hits^2^	Input hits^2^	ChIP/ Input ratio	Repeat type^3^
>10000 ChIP hits (>1%) AND ChIP/Input ratio >1	CL3	42004	38140	1.10	TCAST satellite DNA^4^
	CL4	31220	27988	1.12	TCAST satellite DNA^4^
	CL10	23779	14796	1.61	28S rDNA-like
	CL12	28617	19621	1.46	*T*. *castaneum* LINE-1 element ORF2 protein (LOC658088)
	CL13	42765	26017	1.64	18S rDNA-like
	CL19	12573	9903	1.27	Cast6 satellite DNA^5^
	CL20	10166	7557	1.35	DNA transposon (Helitron)-like
	CL48	10565	5052	2.09	tandem repeat
>100 ChIP hits (>0.01%) AND ChIP/Input ratio >2	CL141	488	158	3.09	*T*. *castaneum* non-LTR retrotransposon SARTTc1
	CL143	460	192	2.40	*T*. *castaneum* non-LTR retrotransposon SARTTc1
	CL169	495	238	2.08	*T*. *castaneum* non-LTR retrotransposon SARTTc3
	CL198	210	99	2.12	DNA transposon (Polinton)-like
	CL234	402	158	2.54	LTR retrotransposon (Copia)-like
	CL253	288	110	2.62	*T*. *castaneum* non-LTR retrotransposon SARTTc3
	CL264	337	145	2.32	*T*. *castaneum* non-LTR retrotransposon SARTTc1
	CL270	732	342	2.14	5S rDNA
	CL298	296	137	2.16	non-LTR retrotransposon (Jockey)-like
	CL336	177	70	2.53	*T*. *castaneum* non-LTR retrotransposon SARTTc3
	CL391	1591	602	2.64	unknown
	CL394	442	85	5.20	*T*. *castaneum* uncharacterized LOC107399056. partial mRNA
	CL399	202	61	3.31	*T*. *castaneum* non-LTR retrotransposon SARTTc1
	CL469	145	53	2.74	*T*. *castaneum* non-LTR retrotransposon SARTTc1
	CL611	216	78	2.77	*T*. *castaneum* tigger element-derived protein 4 (LOC103314401)
	CL669	118	58	2.03	unknown
	CL711	174	78	2.23	*T*. *castaneum* non-LTR retrotransposon SARTTc3
	CL797	115	41	2.80	*T*. *castaneum* mobile element jockey-like (LOC103314666)
	CL942	133	39	3.41	unknown
	CL992	118	34	3.47	*T*. *castaneum* non-LTR R2 retrotransposon

^1^Clustering of 270200 randomly selected WGS Illumina reads, corresponding to 0.2x genome coverage, was performed using RepeatExplorer2 pipeline [31].

^2^ ChIP and Input hits values were generated using one million cCENH3-ChIP and Input reads in ChIP-seq Mapper analysis [7]. ChIP enrichment was calculated using the top 1000 most repetitive WGS clusters obtained by RepeatExplorer2 clustering.

^3^ Repeat type was determined by BLAST searching against GIRI Repbase and NCBI GenBank database.

^4^ Ugarković *et al*. [25]

^5^ Pavlek *et al*. [28]

### Repeat classification of cCENH3-associated DNA sequences

Among cCENH3-associated tandem repeats, the clusters CL3 and CL4 dominate in terms of ChIP hits numbers (Table 1). These two clusters belong to the *T*. *castaneum* major satellite DNA TCAST, based on 360 bp long monomers [25]. In addition to being highly abundant, TCAST is a very heterogeneous satellite family composed of five subfamilies mutually divergent up to 30% [28]. The RepeatExplorer2 analysis assorted the TCAST satellite into four clusters, CL1-CL4, which cumulatively comprise 13.8% of the genome (Fig 5, Table 2). According to ChIP-Seq Mapper analysis, the clusters CL3 and CL4 were found enriched 10% and 12%, respectively, while the clusters CL1 and CL2 showed the ChIP/Input ratios of 0.84 and 0.90, respectively (Table 2). We analyzed the proportion of individual TCAST subfamilies defined by Pavlek *et al*. [28] in the CL1-CL4 clusters (Table 2), and we found that none of the four clusters corresponds specifically to any of the TCAST subfamilies. For instance, the cluster CL3 includes mainly the reads associated to the subfamilies 1 and 4, but it also comprises the reads associated with subfamilies 2, 3 and 5 (Table 2). Similarly, CL4 is mostly based on the subfamily 3 reads, but it also harbors the reads sharing similarity with other subfamilies (Table 2). It is possible that the clusters cannot be attributed to the individual subfamilies because they were generated from the reads of submonomeric length (151 bp of ~360 bp). Since we did not find either a motif or any specific trait that would strictly differentiate CL3 and CL4 reads in comparison to CL1 and CL2 reads, we presume that all five TCAST subfamilies participate in the centromeric chromatin.

**Table 2:** Contribution of the TCAST satellite subfamilies to four most abundant WGS clusters.

Cluster^1^	Genome proportion^1^	cCENH3-ChIP enrichment^2^	TCAST subfamilies^3^ proportions in the cluster				
			Subfamily 1	Subfamily 2	Subfamily 3	Subfamily 4	Subfamily 5
**CL1**	4.5%	0.84	7.18%	0.18%	15.27%	35.50%	41.25%
**CL2**	4%	0.90	71.32%	22.33%	0.14%	5.47%	0.67%
**CL3**	3.1%	1.10	47.12%	7.58%	2.20%	39.17%	3.88%
**CL4**	2.2%	1.12	0.03%	0.58%	82.12%	9.06%	8.14%

^1^ Clustering of WGS Illumina reads and their estimated genome proportions were obtained by RepeatExplorer2 analysis [31].

^2^ ChIP enrichment for cCENH3 protein was calculated by performing ChIP-seq Mapper analysis [7]. The values represent the ratio of cCENH3-ChIP hits and Input hits.

^3^ Satellite DNA TCAST classification into five subfamilies is based on Pavlek *et al*. [28]. The subfamily consensus sequences were used in RepeatExplorer2 clustering to determine the proportions of satellite subfamilies within each cluster.

**Fig 6:**
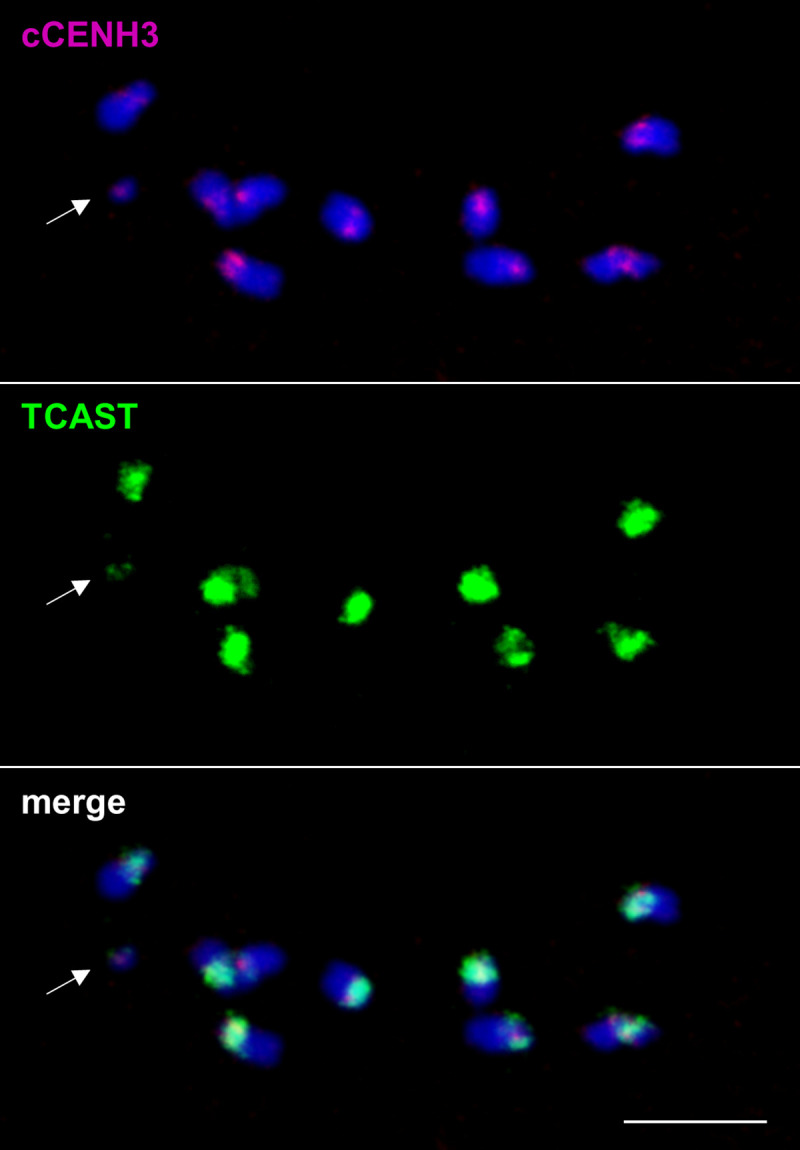
Co-localization of cCENH3 protein and TCAST satellite DNA on *Tribolium castaneum* meiotic chromosomes. Combined IF-FISH analysis of *in situ* localization of cCENH3 protein (shown in magenta) and TCAST satellite DNA (shown in green) demonstrated their co-localization at centromeric regions of all *T*. *castaneum* chromosomes. An arrow points to the y_p_ chromosome. Scale bar = 5 μm.

**Fig 7:**
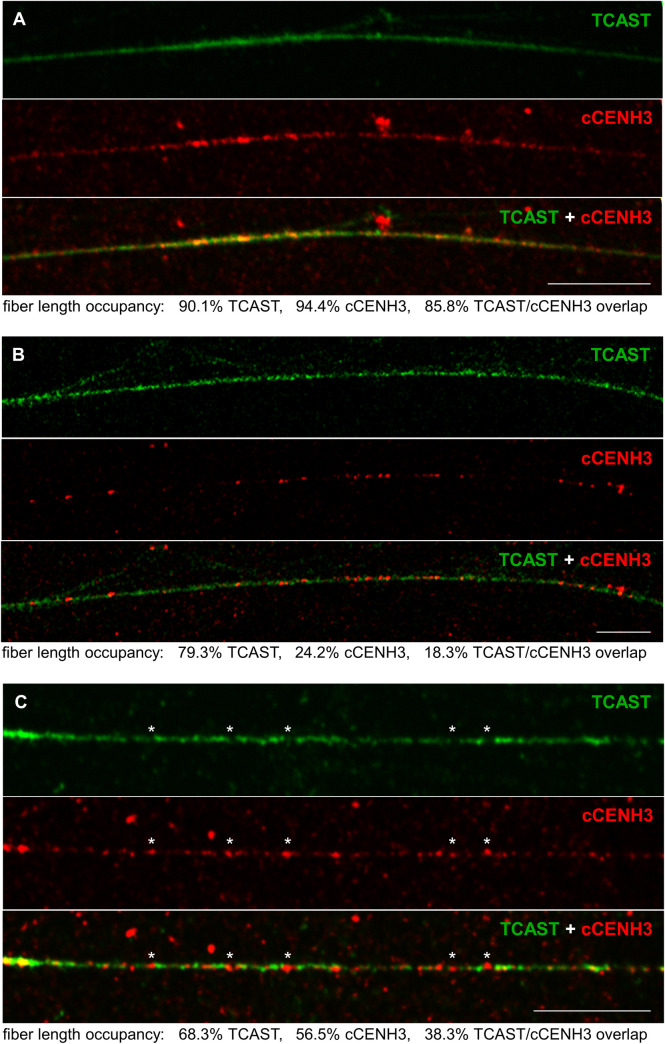
Co-localization of cCENH3 protein and TCAST satellite DNA on *Tribolium castaneum* extended chromatin fibers. By combined IF-FISH analysis, position of TCAST satellite DNA (green signals) was revealed by FISH, while the position of cCENH3 protein (red) was detected by IF. The examples of a fiber with prominent TCAST-cCENH3 overlapping (A), a fiber mostly deprived of cCENH3 signal (B), and a fiber with asterisks-mark positions of cCENH3 signals which do not coincide with TCAST satellite signals (C) are shown. The percentages of the chromatin fiber length occupied by TCAST and cCENH3 signals are indicated under the panels. Scale bar = 10 μm.

Although the reads belonging to TCAST satellite dominate by number in the ChIP sample, based on previously established TCAST localization in the heterochromatic (peri)centromeric blocks of *T*. *castaneum* chromosomes [25], we expected that TCAST would show higher enrichment for cCENH3 than it did in our ChIP experiment. Therefore, to explore *in situ* interrelation between cCENH3 and TCAST satellite DNA, we performed combined IF-FISH using the mixed DNA probe representative of all five TCAST subfamilies. First, IF-FISH experiments were conducted on *T*. *castaneum* metaphase chromosomes (Fig 6). In spite of the fact that the intensity and the extent of TCAST signals on metaphase chromosomes oscillate from very strong at majority of autosomes to discrete at the y_p_ chromosome, IF-FISH confirmed that cCENH3 and TCAST signals coincide at centromeric regions of all chromosomes. The expanse of the FISH signals indicates that TCAST arrays outspread beyond cCENH3 domains, suggesting their presence also in the pericentromeric regions. Additionally, the TCAST signal outspread beyond the cCENH3 domains could potentially be influenced to some degree by the methodology itself as the use of acetic acid in IF-FISH chromosome preparation reduces the cCENH3 signal strength, while the FISH signal amplification procedure could result in the TCAST signal overestimation. Since resolution of highly condensed chromatin at metaphase chromosomes does not provide insight into linear organization of centromeric chromatin constituents, we applied IF-FISH to extended chromatin fibers. Optical mapping on extended chromatin fibers revealed long arrays of TCAST satellite that were also largely occupied by cCENH3 protein (Fig 7A). The quantification of the 15 chromatin fibers with high presence of both TCAST and cCENH3 signals showed that TCAST/cCENH3 signal overlaps cover between 62% and 88% of the measured fibers’ length (S5 Appendix). We observed, nevertheless, that at some continuous TCAST arrays cCENH3 is loosely distributed (Fig 7B). cCENH3-poor regions could be “linkers” between cCENH3-rich domains observed as bead-like patterns at prometaphase chromosomes (Fig 3C and 3D). Alternatively, they might come from pericentromere with potentially sparse cCENH3 domains. Additionally, in some segments of chromatin fibers we also noticed sporadic distribution of cCENH3 signals that do not co-localize with TCAST signals, but reside in the gaps within TCAST arrays (Fig 7C). It can be assumed that at these scattered spots cCENH3 nucleosomes are associated with other sequence candidates listed in Table 1.

Within tandem repeat class of sequences, in addition to TCAST, ChIP-seq data revealed enriched cluster CL19 (Table 1) which corresponds to significantly less abundant satellite DNA Cast6. Cast6, being based on a ~180 bp monomer unit and making up to 0.5% of the genome, is one of the *T*. *castaneum* satellites that were proclaimed to be present primarily in euchromatic regions [28]. However, Cast6 occurrence in the centromeric heterochromatin blocks of at least one chromosome [28] suggests that some of its arrays contribute to the centromere chromatin. It has to be stressed that among nine “euchromatic” satellites that have been described in *T*. *castaneum* [28], only Cast6 was cCENH3-enriched, while the others were underrepresented in ChIPped data set showing enrichment factors between 0.44–0.73. CL48 is another tandem repeat enriched in cCENH3 reads (enrichment factor of 2.09, Table 1), that comprises 0.05% of the genome. Composed of average 73 bp long monomers and predominantly present in unplaced singletons, this repeat represents a novel satellite DNA not described in the *T*. *castaneum* genome so far.

In the group of cCENH3-enriched clusters that comprise mobile elements, there are predominant nine clusters which share similarity with *T*. *castaneum* non-long terminal repeat (non-LTR) retrotransposons from the SARTTc family. The SARTTc family includes seven types of retrotransposons (SARTTc1-SARTTc7) with target sequence preference to telomeric TCAGG motif [32]. According to our analysis, five clusters CL141, CL143, CL264, CL399, and CL469, enriched 2.3–3.3 times, are related to the non-LTR retrotransposon SARTTc1 sequence segments, while the clusters CL169, CL253, CL336, and CL711, with enrichment factors 2.1–2.6, are associated with the SARTTc3 sequence (Table 1). The presence of SARTTc sequences in the centromeric chromatin could explain the weak telomeric FISH signals on some of the *T*. *castaneum* chromosomes obtained by cCENH3-ChIPped DNA probe (S4 Fig).

Among rDNA-related clusters, the cluster CL270 shows the highest ChIP/Input ratio of 2.14 (Table 1). Interestingly, this cluster is identical to the 5S rDNA, whose 192 bp long repeat unit consists of a 119 bp long 5S rRNA gene and 73 bp long non-transcribed spacer (NTS) region. Although centromeric regions are not typical rDNA locations, ChIP enrichment suggests that a portion of the 5S rDNA arrays is drafted into centromere function. Besides tandem repeats, mobile elements and rDNA-like sequences, ChIP-seq analysis implies that other sequence candidates, whose status we have not been able to determine, are likely to be centromeric (Table 1). Even though their contribution might be less prominent in terms of sequence abundance, they manifest the high diversity of DNA sequence composition at *T*. *castaneum* centromeres.

## Discussion

The red flour beetle *T*. *castaneum* with unassembled 20% of the genome corresponding to (peri)centromeric regions [23] certainly represents a challenging and compelling model organism for centromere biology research. In this work we ascertained cCENH3, the centromere-specific histone H3 of *T*. *castaneum*, and utilized it to determine architecture and characterize DNA composition of *T*. *castaneum* centromeres.

We identified the gene ID TC012577 from the current *T*. *castaneum* genome assembly Tcas5.2 [24] as the CenH3 homologue, and its coding sequence is identical to a candidate computationally predicted by Drinnenberg and coauthors [6]. By demonstrating cCENH3 chromosomal position, we validated cCENH3 experimentally as an authentic centromeric variant of histone H3. In this way it was confirmed that the prediction of CenH3 protein using defined bioinformatics criteria [14] was accurate and reliable in the *T*. *castaneum* case. CenH3 proteins differ from the canonical H3 foremost in N-tail domain, and CenH3 N-tail domain often extends over 100 amino-acid residues, as in *Drosophila melanogaster* CID [33], *Caenorhabditis elegans* HCP-3 [34] or *Saccharomyces cerevisiae* CSE4 [35]. *T*. *castaneum* cCENH3 is a rather small protein of 125 aa, having N-tail domain even shorter than the canonical H3 (24 aa *versus* 40 aa). Despite the shortness of NH_2_-terminal tail, we successfully produced an antibody that specifically recognizes cCENH3 and discriminates it from the canonical H3.

The repertoire of *T*. *castaneum* centromeric DNA sequences, which we obtained by cCENH3-ChIP-seq, includes a variety of repetitive DNAs. Notably, all of these sequences, regardless of a repeat type they belong to, are predominantly found in the unplaced scaffolds and singletons related to the unassembled part of the current Tcas5.2 genome assembly. Association with the unassembled genome fraction might indicate that these sequences belong to centromeric regions that have not yet been delineated for any of *T*. *castaneum* chromosomes. Not surprisingly, the major centromeric DNA constituent is the TCAST satellite that comprises one-sixth of the genome [25]. According to optical mapping on metaphase chromosomes and extended chromatin fibers, TCAST constitutes very long arrays that also include cCENH3 domains. Cytological experiments showed, however, that TCAST is not limited to the centromeres, and its arrays stretch beyond the cCENH3 signals. TCAST presence in the pericentromere could partly be a reason why its enrichment in cCENH3-ChIP experiments was lower than expected. Furthermore, it has been known for a long time that at centromeres with underlying highly abundant repeats, CenH3 nucleosomes interact generally only with a fraction of those repeats [36,37], and such a partial occupancy can diminish their relative ChIP enrichment. In the recent study of *D*. *melanogaster* centromeres, its most copious centromeric repeat *Prodsat* revealed lower CenH3-enrichment in comparison to AATAG repeat, although *Prodsat* was seven times more abundant in the IP fraction [38], and the authors stressed that not only enrichment, but also the abundance of a sequence has to be regarded when estimating its centromere potential. In the model plant species *Arabidopsis thaliana* functional centromeres assemble on the most homogeneous and evolutionary youngest specific subset of CEN180 repeats, mostly excluded from the genome assembly [39]. Similarly, in human centromeric chromatin dominate two dimeric alpha-satellite DNA units, mutually highly diverged, but both being highly homogenized and younger than the rest of the alpha-satellite variants [40]. *T*. *castaneum* TCAST has been described as a very divergent satellite DNA compiled from five subfamilies [28], but in our study we have not found an evidence that cCENH3-containing nucleosomes have any preference for a unique subfamily or a subset of TCAST monomers. According to FISH analysis performed by Pavlek and coauthors [28], all five TCAST subfamilies coincide at the centromeric regions of all chromosomes. Computational analysis of higher order repeat (HOR) structures in *T*. *castaneum* disclosed TCAST-incorporating HORs found solely in unplaced scaffolds and singletons; particularly interesting is the fact that the TCAST monomers in those HORs are very heterogeneous, come from different subfamilies and are combined with extraneous sequence elements [41]. Our ChIP results corroborate previous cytogenetic TCAST localization [28] and bioinformatic HOR analysis [41], and suggest that functional *T*. *castaneum* centromeres are not built upon an exclusive fraction of TCAST satellite. Instead, it is more likely that they comprise different variants/subfamilies of the major satellite DNA intermingled with other DNA sequences.

Satellite DNAs are among the most frequent DNA constituents of functional centromeres [42], and therefore it is not unexpected that in cCENH3-ChIP data set, beside the major satellite DNA TCAST, we also found enriched other satellite DNAs. However, those satellites are less abundant by an order of magnitude or even two in comparison to TCAST. Thirteen satellite DNA families whose genome abundance differ ~10–1000 times have been recorded in the pea centromeres [7], confirming that tandem organization is favorable, while abundance of a tandem repeat could be less determinative criteria for centromeric assortment. In addition to satellites, retroelements can be equally important or even dominant centromere components [43], and contribution of retrotransposons to functional centromeres have been documented in different animal, plant and fungal lineages [43–47]. In *T*. *castaneum*, in the group of different cCENH3-associated transposable elements we found prevalent non-LTR retrotransposons SARTTc. Notwithstanding SARTTc elements were reported as telomeric retrotransposons that intermingle with TCAGG repeats in *T*. *castaneum* “composite” telomeres [32], we mapped them in unassembled scaffolds in the vicinity of (TCAGG)n arrays that are relatively short (100–400 bp) and might represent heterochromatic interstitial telomeric sequences (ITS) within centromeric regions. It has been assumed that ITSs at (peri)centromeres are mostly remnants of telomere-mediated DNA repairs and chromosomal rearrangements [48], but for different *Solanum* species including tomato, potato and eggplant, it was revealed that some ITS subfamilies expanded into functional centromeres [49]. Besides satellite repeats and retrotransposons, in the *T*. *castaneum* centromeric chromatin we also detected rDNA-associated sequences, and the most prominent of them is 5S rDNA with its NTS region. rDNA tracts can serve as an origin of satellite repeats [50,51], and centromerically located satellite DNA partially derived from 5S rDNA was described, for instance, in the frog *Physalaemus cuvieri* [52]. Remarkably, in the switchgrass *Panicum virgatum* it was recently shown that one of its centromeres propagated the exact 5S rDNA unit with its completely conserved 5S rRNA gene sequence and NTS region into functional centromeric DNA, without obstructing the fundamental function of the 5S rRNA genes [53]. We believe a similar scenario could be assumed in *T*. *castaneum*. Its centromeres, compiling different kinds of repetitive sequences, confirm that centromeric regions can be quite permissive regarding their DNA content, and that the presence of one highly abundant and dominant satellite DNA does not exclude or prevent other repetitive sequences to contribute. It can be even speculated that a widely outspread, abundant satellite DNA allows and/or fosters intrusion of different sequences whose locations are not primarily or exclusively centromeric. It is questionable, though, whether these intrusive elements are indeed epigenetically favored or simply tolerated given their scarcity in the centromeric regions.

What makes *T*. *castaneum* centromeres exceptional is their unusual extent. We estimated that *T*. *castaneum* centromeres, ranging in size from 2.3 to 15.8 Mb, comprise over 40% of the chromosome length. In comparison, the centromeric regions in *D*. *melanogaster*, the insect of comparable genome size (180 Mb *vs*. 200 Mb), range between 100 and 170 kb [43]. To the best of our knowledge, *T*. *castaneum* centromeres are the longest insect regional centromeres described so far. Interestingly, in the red imported fire ant *Solenopsis invicta* unusually long centromeres, on average spanning one third of chromosomes’ length (3.6 Mb), have also been observed [54]. In addition to the remarkable length, *T*. *castaneum* centromeres are marked by their metapolycentric structure, characterized by multiple CenH3-domains stretching along a large chromosome region that forms an extended, yet single primary constriction. Neumann and colleagues [7] introduced the term of metapolycentricity to describe such an unusual type of centromeres evidenced in the two closely related plant genera *Pisum* and *Lathyrus* [7,8]. In comparison to *T*. *castaneum*, the peas’ genomes are 20–30 times bigger and their chromosomes are remarkably longer, so it does not surprise that the pea metapolycentric centromeres are notably larger ranging in size from 69 to 107 Mbp [7]. Notwithstanding the significant difference in centromere size, the peas’ and *T*. *castaneum* centromeres share the bead-like pattern, a hallmark of metapolycentric structure.

The presence of metapolycentricity in very distant eukaryotic species suggests its convergent evolution, but the foundation of metapolycentricity remains an open question. In the pea species with metapolycentric chromosomes, CenH3 proteins are encoded by two paralogous genes that are equally expressed, and the two CenH3 proteins co-localize at the same domains of extended centromeres [7,8]. Nevertheless, Neumann and coauthors [8] warn that the potential dosage effect of *CenH3* duplication cannot be solely responsible for such a drastic centromere expansion. *T*. *castaneum* cCENH3 protein is encoded by a single gene, and we found no evidence of additional *CenH3* copies, which makes us conclude that metapolycentricity in *T*. *castaneum* arose through mechanisms unrelated to *CenH3* duplication. Different chromosomal rearrangements, especially Robertsonian centric fusions, can lead to the enlarged centromere size. One of the most illustrative examples is an unusually long, bead-like kinetochore region of chromosome C3X in Indian muntjac, the mammal whose small karyotype (2n = 6♀,7♂) evolved through numerous linear centromere-telomere fusions from the acrocentric 2n = 70 ancestral karyotype [55,56]. As the *T*. *castaneum* karyotype is based on the Coleoptera prevalent diploid number 2n = 20, it is not very plausible that its metapolycentromeres were generated via chromosomal fusions. In our opinion, the expansion of *T*. *castaneum* centromeres could be most convincingly explained by centromere drive hypothesis [17,18,57]. According to the centromere drive model, “stronger” centromeres, i.e. those that segregate more successfully to the egg during female meiosis, provide more microtubule attachment sites by recruiting more centromeric proteins, and enhanced recruitment of centromeric nucleosomes could be driven by centromeric satellite expansion [17]. In a mouse model system it was shown that centromeres with more centromere-competent satellite repeats indeed engage more CENP-A, thus gaining a transmission advantage in the female germline [58]. We hypothesize that the highly repetitive *T*. *castaneum* metapolycentric regions can be evolutionary favored due to increased capacity for promoting cCENH3 chromatin expansion, which might ensure a selective advantage in the race to reach the oocyte. Similarly, long primary constrictions in the *Solenopsis* fire ants coincide with the position of the major, high-copy satellite DNA according to FISH analysis, and centromere drive was proposed to propel centromere elongation [54]. Although CenH3 localization was not examined so there is no exact data on fire ants’ centrochromatin structure, it is possible that metapolycentricity among insects, especially those with highly abundant centromeric satellites, is not unique to *Tribolium*.

Morphology of metapolycentric chromosomes suggests an intermediate state between monocentric and holocentric chromosome architecture [59]. It has been proposed that holocentricity has evolved at least 13 times among eukaryotes [4]; nevertheless, the evolutionary relationship between holocentricity and monocentricity remains an enigma. Assuming monocentricity as the initial state, Drinnenberg and coauthors [6] revealed independent transitions to holocentricity in at least four lineages of insects. Significantly, 13 of the 15 analyzed holocentric insect species have lost CenH3, and it was theorized that CenH3 loss was preceded by monocentricity-to-polycentricity architectural changes [6,60]. It is tempting to speculate that *T*. *castaneum*, which engages CenH3 in its extended metapolycentromeres, could represent an intermediate between monocentricity and polycentricity in insects. However, further extensive analyses are required to test this speculative hypothesis.

The beetle *T*. *castaneum* with its metapolycentric chromosomes whose centromeres are founded on one, genome-prevailing satellite DNA is a dare *par excellence* for the correct centromere assembly. For complete understanding how kinetochore sites are designated and maintained in diverse eukaryotic species, the studies of kinetochore assembly in organisms with unusual centromere anatomies are crucial. We believe that *T*. *castaneum*, as the coleopteran model organism, could also be an excellent model to address these questions.

## Materials and methods

### Insect material

All experiments in this work were performed using the highly inbred Georgia 2 (GA2) strain of the red flour beetle *T*. *castaneum*. The initial GA2 strain stock was obtained from USDA-ARS (Manhattan, Kansas, USA), and subsequently maintained as a laboratory culture. The insects were reared in whole wheat flour at 28°C, and sub-cultured every four weeks. In addition to GA2 strain, genomic DNAs isolated from the two additional *T*. *castaneum* strains DE and ES (obtained from Germany and Spain, respectively) were used for checking the nucleotide sequence of the *cCENH3* gene.

### DNA extraction and PCR amplification

Total DNA was isolated from ~50 mg of adult insects (25 individuals) by using DNeasy Blood and Tissue Kit (Qiagen). DNA quantification was done by Qubit fluorometer (Invitrogen) using Qubit dsDNA BR Assay Kit (Thermo Fisher Scientific). Based on the *cCENH3* coding sequence and its flanking regions, the primers cCENH3pr0 (5'- AAGGTAGGATAATGCCGA-3'), cCENH3pr1 (5'-ATGGCCCGTTCTAAG-3'), cCENH3pr3 (5'-TTAACCACCTTCTTTTTCC-3’), and cCENH3pr5 (5'-TTAACCACCTTCTTTTTCCCTCC-3’) were designed to amplify the entire *cCENH3* gene sequence. PCR reaction was performed in 50-μl volume containing 1× Green GoTaq Reaction Buffer (Promega), 2.5 mM MgCl_2_, 0.1 mM of each dNTP, 0.1 μM of each primer and 2 units of GoTaq G2 DNA Polymerase (Promega). PCR program included predenaturation at 94°C for 3 min, 35 cycles od amplification (denaturation at 94°C for 20 s, annealing at 61°C for 20 s, and extension at 72°C for 30 s), and final extension at 72°C for 7 min. PCR products were analyzed in agarose gel electrophoresis, extracted by QIAquick Gel Extraction Kit (Qiagen), and Sanger-sequenced in Macrogen Europe Laboratory (Amsterdam, The Netherlands).

### RNA isolation and reverse transcription

Total RNA was purified from ~55 mg of larvae (20 individuals) by using RNeasy Mini Kit (Qiagen), and additionally treated with RQ1 RNase-Free DNase (Promega). RNA was quality-checked on agarose gel, and quantified by BioSpec-nano Micro-volume UV-Vis Spectrophotometer (Shimadzu). The coding region of the *cCENH3* gene was obtained by RT-PCR amplification using OneStep RT-PCR Kit (Qiagen) and the gene-specific primers cCENH3pr1 and cCENH3pr3. cDNA was synthesized from 250 ng of RNA in 25 μl RT reaction at 50°C for 30 min. Initial PCR activation step at 95°C for 15 min was followed by 35 cycles of amplification (denaturation at 95°C for 30 s, annealing at 61°C for 30 s, and extension at 72°C for 1 min), and final extension at 72°C for 10 min using HotStarTaq DNA Polymerase. Three negative controls were included in the RT-PCR experiment: 1) a control reaction in which reverse-transcriptase activity was inhibited by keeping the reaction on ice and placing it in the thermal cycler only after it had reached 95°C for the HotStarTaq DNA Polymerase activation step, 2) a control reaction in which RNA template was added during the HotStarTaq DNA Polymerase activation step, 3) a blank control without template RNA. RT-PCR *cCENH-3* products were purified using PCR Purification Kit (Qiagen), and Sanger-sequenced with the primers above.

### cCENH3 expression analysis

To examine the expression pattern of the *cCENH3* gene, we used publicly available RNA-seq datasets from Khan *et al*. [29] deposited at the Gene Expression Omnibus (GEO) repository under accession number GSE119739. Sets of transcriptome reads corresponding to embryo 0–5 hr, embryo 5–11 hr, ovary, testis, female carcasses (lacking ovaries), and male carcasses (lacking testes), were separately mapped to the *cCENH3* gene sequence using Bowtie2 with—local and—very-sensitive option implemented in Geneious R11.1.4 (Biomatters, Ltd.). All obtained hits were normalized with CPM method ((counts per million reads mapped) = (hit number/library size)*10^6^) and average values were calculated for different runs and biological replicates (four runs for each replicate; two replicates for testes, three replicates for all the other samples).

### Antibody design and production

Polyclonal IgG antibodies were generated against a synthetic peptide NH_2_-RSKKTPNKKPSSASTSYF-CONH_2_, corresponding to amino acids 3–20 of the N-terminal end of the *T*. *castaneum* cCENH3 protein (Fig 1). Peptide synthesis and immunization of two rabbits were performed by Pineda Antikörper-Service (Berlin, Germany). To monitor the development of the immune response, we tested by Western blotting the preimmune sera as well as the sera samples on the monthly basis during the immunization period. Although the preimmune sera showed certain background of non-specific bands corresponding to higher molecular weight proteins in the Western blot testing (S3 Fig), the polyclonal antibodies to the targeted protein of ~15 kDa were first detected after the 2-month immunization period. After 120 days, the immunization was stopped, and affinity purification of the monospecific IgG fraction from cCENH3-antisera on sepharose columns was performed. Purified monospecific IgG fraction was concentrated 25x using Amicon Ultra-0.5 centrifugal filter device (Merck), and used in all downstream applications.

### Protein extraction and Western blot

125 mg of snap-frozen *T*. *castaneum* adults was ground to a fine powder using mortar, pestle and liquid nitrogen. Immediately after grinding, tissue powder was transferred to 5 ml of ice-cold RIPA buffer (150 mM NaCl, 1% Triton X-100, 0.5% sodium deoxycholate, 0.1% SDS, 50 mM Tris-HCl, pH 8.0) supplemented with 2 mM PMSF and cOmplete Mini EDTA-free protease inhibitor cocktail (Roche). The suspension was homogenized in an ice-cold glass dounce homogenizer, and the cells were disrupted with 15–20 strokes of the pestle. The sample was rotated on an orbital shaker (7 rpm) for 2 h at 4°C. The homogenate was centrifuged for 20 min at 13500 g at 4°C, and the supernatant containing the protein extract was collected, aliquoted to ice-chilled tubes, and stored at -80°C until use. The protein concentration was estimated using the Bradford protein assay. For Western blot, 20 μg of proteins per reaction was resuspended in 15 μl total volume of 1xLaemmli buffer (50 mM TrisHCl pH 6.8, 10% glycerol, 2% SDS, 0.005% bromophenol blue) supplemented with 0.1 M dithiothreitol (DTT). The samples were heated at 37°C for 30 min, and then run on a 4–20% Mini-PROTEAN TGX precast protein gel (Bio-Rad) and transferred onto Amersham Protran Supported 0.2 μm nitrocellulose membrane (GE Healthcare Life Sciences). For probing, the rabbit preimmune serum or the primary anti-cCENH3 antibody, and the horseradish peroxidase (HRP)-linked goat anti-rabbit IgG antibody (Cell Signaling Technology), were used at 1:500 and 1:2000 dilution, respectively, in TBST buffer (20 mM Tris, 150 mM NaCl, pH 7.6, 0.1% Tween 20) supplemented with 5% BSA. The blots were developed using the Pierce ECL Western Blotting substrate (Thermo Scientific) and Amersham Hyperfilm ECL X-ray films (GE Healthcare Life Sciences).

### Cytological preparations

Immunofluorescence (IF), fluorescence *in situ* hybridization (FISH), and IF-FISH experiments were done on metaphase spreads, interphase nuclei and chromatin fibers isolated from the gonads of male pupae. We optimized, however, different protocols for IF, FISH and IF-FISH chromosome and fiber preparations in order to preserve chromosome/chromatin architecture and the antibody’s epitopes, but at the same time allowing the penetration of the DNA probes when FISH performed.

For IF experiments, freshly isolated male gonads were incubated in 10 μg/ml colcemid solution for 1–2 h, then transferred to 0.5% sodium citrate for 5 min. After hypotonic shock, the testes were incubated in pre-warmed 2% paraformaldehyde (PFA) at 37°C for 10 min, then transferred to phosphate-buffered saline (PBS) supplemented with 30 mM glycine for 10 min at RT. After 3x5 min washing in PBS, the tissue was manually disaggregated on a slide in a drop of 0.05 M NaOH, covered with a coverslips and squashed. Slides were then frozen briefly (30–60 s) in liquid nitrogen. Coverslips were removed immediately, and slides were air-dried and stored at -80°C until use. For FISH experiments, freshly dissected testes were incubated in 10 μg/ml colcemid solution (Roche Diagnostics) for 1–2 h at RT. The tissue was subjected to hypotonic shock in 75 mM KCl for 15 min, and then fixed in Clarke’s solution (absolute ethanol:acetic acid, 3:1) for at least 30 min. The testes were macerated on a slide in a drop of 45% acetic acid, squashed and frozen as described for IF slide preparation. Air-dried slides were stored at -20°C until use.

For combined IF-FISH analysis, dissected male gonads were incubated in 10 μg/ml colcemid solution for 1–2 h, and subjected to hypotonic shock in 75 mM KCl for 15 min. The fixation was done in freshly prepared solution of 3.7% PFA, 5% acetic acid and 0.9% sodium chloride at RT for 18 min. After 10 min washing in PBS, the gonads were disaggregated and squashed on a slide in a drop of 0.05 M NaOH, frozen and processed as described for IF slide preparation, being kept at -80°C until use. In all aforementioned cytological preparations, the poly-D-lysine coated slides were used.

The extended chromatin fibers were prepared from dissected testes isolated and washed in PBS supplemented with 1 mM PMSF. Approximately 20 gonads resuspended in 100 μl PBS + 1 mM PMSF were mixed by microtube homogenizer for 30 s, supplemented with 700 μl PBS + 1 mM PMSF, and strained through 100 μm cell strainer. Using Cytospin 4 cytocentrifuge (Shandon, ThermoFisher Scientific), 400 μl of suspension was cytospun for 10 min at 1200 rpm onto coated Shandon Cytoslides (ThermoFisher). After short air-drying, 15 μl of a freshly prepared mild SDS lysis buffer (200 mM Tris-HCl, pH 7.4, 50 mM EDTA, 0.2% SDS, 1 mM PMSF) was added per slide, that was immediately covered with a 18x18 mm square coverslip. After 4–8 min lysis reaction at room temperature, a coverslip was lifted slowly. Air-dried slides were fixed in 2% formaldehyde diluted in PBS for 10 min, and then washed in PBS for 3x5 min. Four drops of Image-iT FX signal enhancer (ThermoFisher Scientific) were applied per slide, and slides were covered by 20x40 mm coverslips and incubated for 30 min at room temperature in a humid chamber. After that, coverslips were removed, and slides were thoroughly rinsed with PBS for 3x5 min, and directly subjected to IF-FISH procedure.

### Immunofluorescence (IF) and fluorescence *in situ* hybridization (FISH)

For IF, slides were rinsed in PBS for 2 min, and then incubated in PBS containing 1% Triton X-100 for 25 min on ice. After 3x5 min washing in PBST (PBS, 0.2% Tween 20), slides were blocked in PBST supplemented with 2.5% BSA and 0.3 M glycine in a moist chamber at 37°C for 1 h. Anti-cCENH3 antibody was diluted 1:400 (for chromosome spreads) or 1:200 (for chromatin fibers) in blocking solution (PBST containing 2.5% BSA), and added to slides which were incubated in a humid chamber overnight at 37°C. Slides were washed 5x5 min with PBST at RT, and then incubated for 1 h at 37°C with Alexa Fluor 594-labeled or Alexa Fluor 488-labeled goat anti-rabbit antibodies (Abcam), diluted 1:1000 in blocking solution. Final washes were performed 4x5 min in PBST, and 1x5 min in PBS at RT. Slides were counterstained in 4′,6-diamidino-2-phenylindole (DAPI) solution for 15 min, then rinsed briefly with distilled water and air-dried. Slides were finally embedded in Mowiol 4–88 mounting medium (Sigma-Aldrich).

For FISH, cCENH3-ChIPped DNA was labeled with Cy3-dCTP (GE Healthcare Life Sciences) using random priming assay. TCAST satellite probe was prepared from the mix of plasmid clones CT8 and CT19 (available upon request) and labelled with biotin-16-dUTP (Roche) by PCR using the primers Tcastan1 (5’-TGTAGGACTAACCATAAGCG-3’) and Tcastan2 (5’-CAATGTTTGAGACGAAGACG3’). Slides were pretreated with RNase A and pepsin, post-fixed by 1% formaldehyde and dehydrated in an ice-cold ethanol series. Slides were denatured in 70% formamide at 70°C for 2 min and hybridized with denatured probes (150 ng of a labeled DNA probe in 15 μl of 60% deionized formamide, 8% dextran sulfate, 1.6xSSC, 20 mM sodium phosphate pH 7.0) overnight at 37°C. Posthybridization washes were done at 37°C in 50% formamide. While the Cy3-labeled cCENH3-ChIPped DNA probe was detected directly after posthybridization washes, the TCAST biotin-labeled probe was visualized with fluorescein avidin D and biotinylated anti-avidin D system (Vector Laboratories) by signal amplification through three layers of fluorophore conjugates and antibodies using the following dilutions: 1:500 fluorescein avidin D, 1:100 biotinylated anti-avidin D, 1:2000 fluorescein avidin D. Preparations were counterstained and embedded as described above.

When IF was followed by FISH, RNase A and pepsin pretreatments were omitted.

### Microscopy and image analyses

Image acquisition was done using a confocal laser scanning microscope Leica TCS SP8 X (Leica Microsystems) equipped with a HC PL APO CS2 63×/1.40 oil objective, 405 nm diode laser and a supercontinuum excitation laser (Leica Microsystems). Images were captured separately for each fluorochrome and processed with ImageJ [61] and Adobe Photoshop (CS5).

In order to estimate the relative size of *T*. *castaneum* centromeres, the extent of cCENH3 signals was measured. For quantification of the signal lengths, maximum intensity projections of z-stacked images were analyzed using “Measure” tool in ImageJ with normalization of scale length across different images. The lengths of the chromosomes and associated centromeres were measured separately for each color channel that was converted to grayscale. Due to different degree of chromosome condensation, centromere sizes were examined using centromere to chromosome length ratios. The chromosomes ch2, ch3, ch4, and y_p_, which can be differentiated from the other chromosomes by their size and centromere position, have been singled out for individual analysis. More than 30 chromosome spreads with accompanied non-overlapping chromosomes were analyzed. Measurement of FISH signals obtained by cCENH3-ChIPped DNA probe was performed on 10 spreads in the same manner described above for the cCENH3 measurement. The average value was calculated from ratios of cCENH3-ChIPped DNA signal length to chromosome length obtained for all chromosomes.

Quantification of cCENH3 and TCAST signal interrelation on extended chromatin fibers was done by examining 15 selected regions where cCENH3 and TCAST showed evident co-localization. The z-stacked images were first adjusted for regions of interest in order to minimize background interference. The same default threshold was then applied in ImageJ on grayscale channels for each image. Manual spline fitted lines were drawn on chromatin fibers and “Plot profile” tool was used to obtain values of pixel intensities along the selected line. Presence or absence of each signal was evaluated by extracting values that were higher or equal to 0, respectively, followed by comparison of the degree of two signals’ overlap. The scatter plots were done in GraphPad Prism version 8.

### Native chromatin isolation and chromatin immunoprecipitation (ChIP)

750 mg of snap-frozen adult insects were ground to a fine powder and resuspended in 10 ml of ice-cold chromatin isolation buffer, CIB (15 mM Tris HCl pH = 7.5, 60 mM KCl, 15 mM NaCl, 0.34 M sucrose, 0.15 mM spermine, 0.5 mM spermidine) supplemented with cOmplete Mini EDTA-free protease inhibitor cocktail (Roche), 2 mM PMSF and 0.5% Triton X-100. The suspension was homogenized in an ice-cold glass dounce homogenizer and strained through 40 μm cell strainer. The isolated nuclei were centrifuged at 2500 g for 5 min at 4°C, rewashed in CIB, centrifuged again, and resuspended in 1.5 ml CIB supplemented with 1 mM CaCl_2_ and 4 mM MgCl_2_. The chromatin was digested with 200 U MNase (Thermo Scientific) for 30 min at 37°C, and the reaction was stopped by adding EDTA to a final concentration of 10 mM. After centrifugation at 8000 g for 5 min at 4°C, the pellet containing chromatin was resuspended in 1.2 ml of PBS supplemented with 0.5 M NaCl. After rotation on an orbital shaker (7 rpm) for 2 h at 4°C, the sample was centrifuged for 15 min at 15000 g at 4°C. The supernatant containing digested chromatin of preferentially mononucleosomal length was saved, and kept at -80°C until use.

For ChIP assay, first we tested the two commercial ChIP kits, Pierce Magnetic ChIP Kit (Thermo Scientific) and Dynabeads Protein A Immunoprecipitation Kit (Invitrogen), but with them we obtained unspecific immunoprecipitation presumably due to insufficiently stringent washing with commercial washing buffers. Therefore, using Dynabeads Protein A, we modified the protocol as follows. In short, Dynabeads were pre-blocked with BSA, and soluble chromatin was pre-cleared with pre-blocked Dynabeads in ChIP buffer (16.7 mM Tris-HCl pH 8.1, 1.2 mM EDTA, 0.167 M NaCl, 0.01% SDS, 1.1% Triton X-100) supplemented with cOmplete Mini EDTA-free protease inhibitor cocktail (Roche) and 2 mM PMSF, by rotation on an orbital shaker (7 rpm) for 1 h at 4°C. Pre-cleared chromatin was separated on a magnetic rack, and 5% was reserved as an input control sample. In ChIP experiment we tested two different anti-cCENH3 antibody amounts per reaction: 5 μg (as a typical amount) and 8 μg (as a Dynabeads Protein A maximum binding capacity). 30 μl of pre-blocked Dynabeads per reaction were mixed with 5 μg / 8 μg anti-cCENH3 antibody diluted in 120 μl Ab Binding & Washing Buffer (Invitrogen), and antibody binding was done by rotation for 30 min at 4°C. As negative controls, 5 μg of normal rabbit IgG (Cell Signaling Technology) and a mock control (a sample without antibody) were included in each ChIP experiment. The Dynabeads-antibody complexes were separated on a magnetic rack, and 230 μl of pre-cleared chromatin (~4 μg) was added. After 2-hour incubation at 4°C with rotation, the precipitated immunocomplexes were washed in a series of buffers (5 min each wash): 3 times low salt buffer (0.15 M NaCl, 20 mM Tris-HCl pH 8.0, 2 mM EDTA pH 8.0, 0.1% SDS, 1% Triton X-100), 3 times high salt buffer (0.5 M NaCl, 20 mM Tris-HCl pH 8.0, 2 mM EDTA pH 8.0, 0.1% SDS, 1% Triton X-100), once LiCl buffer (0.25 M LiCl, 10 mM Tris-HCl pH 8.0, 1 mM EDTA pH 8.0, 1% sodium deoxycholate, 0.1% Triton X-100), twice TE buffer pH 8.0. Chromatin was eluted from the Dynabeads with 2x100 μl of elution buffer (1%SDS, 0.1M NaHCO_3_) after 2x15 min incubation at 55°C. After RNase A / proteinase K treatment, DNA from ChIP and input samples was extracted using QIAquick PCR Purification Kit (Qiagen). In S6 Appendix we showed the comparison of the subsequent ChIP-Seq analyses using 5 μg and 8 μg anti-cCENH3 antibody in ChIP experiments, as well as the comparison between ChIP experiments performed by using commercial washing buffers and prepared low/high salt buffers. Unlike washing with prepared low/high salt buffers, using commercial washing buffers did not yield any difference between cCEN3-ChIP and input samples, and we concluded that the washing with commercial buffers was not stringent enough. ChIP experiments using different amounts of anti-cCENH3 antibody produced congruent results, and the data for the ChIP experiment with 5 μg anti-cCENH3 were used in the following analysis.

### ChIP-seq data analysis

Following the strategy described by Neumann and coauthors [7], we evaluated the enrichment of repetitive sequences in the ChIP-seq data by using the ChIP-Seq Mapper tool at the Galaxy web-server (https://repeatexplorer-elixir.cerit-sc.cz/galaxy/). The workflow of the ChIP-Seq analysis is shown in S5 Fig. The ChIP-Seq Mapper performs BLASTN similarity search of ChIP reads and control input reads (DNA isolated from the chromatin aliquot prior to ChIP experiment) against repeats’ database used as a reference. Repetitive DNA reference database is obtained by the RepeatExplorer2 tool that performs similarity-based clustering and groups short unassembled WGS reads into repeat clusters [31]. The ChIP enrichment for individual WGS repeat clusters is calculated based on ChIP/Input hit ratio. Sequencing of cCENH3-ChIPped DNA and input DNA control, as well as *T*. *castaneum* whole genome sequencing (WGS) was performed on the Illumina HiSeq2500 platform (Admera Health, USA) resulting in 10904694, 9232324 and 9664142 paired-end reads, respectively. By RepeatExplorer2 tools available at Galaxy web-server (https://repeatexplorer-elixir.cerit-sc.cz/galaxy/), FASTQ reads were preprocessed using cutadapt filtering, quality-filtering (95% of bases equal to or higher than the quality cutoff value of 10), trimming to 151 nt, interlacing, and random sampling (different random number generator seeds tested) to scale down large data sets. To generate repetitive DNA reference database, from 9290920 preprocessed WGS reads, 270200 paired-end reads were randomly selected to ensure low genome coverage (0.2x). Repeat identification by graph-based sequence clustering was performed using RepeatExplorer2 pipeline [31] that recommends low-pass genome sequencing corresponding to 0.01–0.50x genome coverage because in genome skimming only the reads derived from the repetitive regions can produce multiple similarity hits and group into the clusters of frequently overlapping sequences. Also, the repetitiveness of the analyzed genome limits the number of reads that can be analyzed with this pipeline. For highly repetitive genome of *T*. *castaneum*, 0.2x genome coverage by 270200 randomly selected reads was determined as an optimal sample size to be processed. RepeatExplorer2 analysis resulted in 21564 WGS clusters (Fig 5), ranked according their genome proportion. Although the genome proportions for cluster CL167 and onwards were estimated lower than 0.01%, in the ChIP-Seq Mapper analysis we examined the first 1000 clusters. From 6623300 and 3828990 preprocessed reads obtained for cCENH3-ChIPped DNA and input DNA, respectively, for each data set three random sampling subsets were generated comprising 1000000, 500000 or 250000 reads. We performed ChIP-Seq Mapper analysis for the top 1000 WGS repeat clusters testing different bit score thresholds (30, 90, 150). As the results of ChIP-Seq Mapper analysis from three different randomly sampled subsets of ChIP/Input reads were consistent (S7 Appendix), only the data for the 1000000 reads analysis were presented. Using Geneious R11.1.4 software (Biomatters, Ltd.), the sequences of cCENH3-ChIP-enriched clusters were mapped against the current *T*. *castaneum* genome assembly Tcas5.2 [24], comprising ten chromosome/linkage groups (LG) and the unassembled sequence represented as 305 unplaced scaffolds (GenBank accessions DS497665-DS497969) and 1848 unplaced singletons (GenBank accessions GG694051-GG695898). Enriched clusters were also subjected to BLAST search against NCBI GenBank database [62], using different BLAST algorithms (megablast, discontiguous megablast, and blastn). CENSOR tool was used to search GIRI Repbase [63].

## Accession codes

The cCENH3 nucleotide sequence has been deposited in NCBI GenBank under the accession number MT043459. Raw Illumina reads from ChIP-seq experiment have been deposited in the Sequence Read Archive under the study accession number PRJNA606031.

## Supporting information

S1 Fig Alignment of *cCENH3* coding sequence and *cCENH3* cDNA. 378 bp long *cCENH3* coding sequence (gDNA) was amplified from genomic DNAs isolated from three different *T*. *castaneum* strains (GA2, DE, and ES). Complementary DNA (cDNA) was synthesized from *cCENH3* transcript by RT-PCR using primers specific for *cCENH3* gene. Identical nucleotides are indicated by a dot, and only one synonymous substitution is present in ES strain. The cCENH3 protein translation is presented below the nucleotide alignment.
(TIF)

S2 Fig Agarose gel electrophoresis of RT-PCR amplification of *cCENH3* mRNA. Reverse transcription PCR amplification was done on total RNA isolated from *T*. *castaneum* larvae by using the primers specific for the *cCENH3* gene. The three negative controls included reactions: (1) without RT step, (2) with template RNA added after RT step, (3) RT-PCR without template RNA. M lane represents a 100-bp-size marker ladder.
(TIF)

S3 Fig Western blot of the cCENH3 antibody on *Tribolium castaneum* protein lysate. *T*. *castaneum* whole protein extract was fractionated by SDS-PAGE under denaturing conditions and transferred to nitrocellulose membrane. The membrane was subjected to Western blot analysis. (1) Testing of the rabbit preimmune serum. (2) Testing of the monospecific IgG fraction purified from the rabbit immunoserum after 120 days of immunization with the cCENH3-specific peptide (NH_2_-RSKKTPNKKPSSASTSYF-CONH_2_) revealed a ~15 kDa signal which is consistent with the expected molecular weight of cCENH3 protein. Molecular weights of protein sizes are indicated in kDa.
(TIF)

S4 Fig Fluorescence *in situ* hybridization with cCENH3-ChIPped DNA. DNA immunoprecipitated by using cCENH3 antibody (cCENH3-ChIPped DNA) was Cy3-labelled and hybridized to *T*. *castaneum* chromosomes. cCENH3-ChIPped DNA (pseudocolored in green) hybridizes to the centromeric regions of all chromosomes (counterstained in DAPI). Scale bar = 5 μm.
(TIF)

S5 Fig The workflow of cCENH3-ChIP-seq analysis. DNA sequences enriched for cCENH3 were identified following the strategy introduced by Neumann *et al*. [7]. First, the repetitive DNA reference database is formed by low-pass Illumina sequencing and RepeatExplorer2 similarity-based clustering of WGS unassembled reads. Chromatin immunoprecipitation (ChIP) was performed using the cCENH3 antibody. Immunoprecipitated DNA was Illumina sequenced, as well as DNA obtained from the chromatin preparation prior to ChIP (Input). ChIP and Input reads were mapped to the WGS clusters, and the cCENH3-enriched clusters were determined based on ChIP/Input reads elevated ratio.
(TIF)

S6 Fig cCENH3-ChIP-Seq Mapper analysis. ChIP-Seq Mapper analysis based on one million cCENH3-ChIP and one million Input Illumina reads mapped to the top 1000 WGS *T*. *castaneum* repeat clusters obtained by RepeatExplorer2 analysis. (**A)** ChIP-Seq Mapper plot for the top 1000 WGS *T*. *castaneum* clusters analyzed for cCENH3 enrichment. The red line marks the mean ratio between ChIP and Input hits, and the clusters above red line show >2-fold enrichment for cCENH3. **(B)** List of 37 out of the top 1000 *T*. *castaneum* WGS clusters showing cCENH3-ChIP/Input ratio >2.
(TIF)

S1 Table Estimation of *Tribolium castaneum* centromere sizes. (PDF)

S1 Appendix BLASTP search against the *Tribolium castaneum* OGS3 database using histone H3 protein sequence as a query. Distribution of 13 BLAST hits on the H3 query sequence is shown by the graphic alignment. The sequences in red represent H3 matches, while the potential CenH3 candidate (TC012577-PA) is marked in magenta. The matches in black represent partial and less significant hits. The alignments between H3 and all 13 matches are listed below, including the alignment score and expected values, and percentage of query/subject identities and positives.
(PDF)

S2 Appendix RNA-seq data on *cCENH3* expression in different life stages and tissues of *Tribolium castaneum*. Normalized expression of cCENH3 gene in *T*. *castaneum* embryos (0–5 hr and 6–11 hr), ovary, testis, female and male carcasses is based on publicly available RNA-seq datasets from Khan *et al*. [29]. Hits were normalized with CPM method ((counts per million reads mapped) = (hit number/library size)*10^6^). Average values were calculated for separate runs and replicates.
(XLSX)

S3 Appendix Related to Fig 4. Source data for *Tribolium castaneum* centromere size estimation.
(XLSX)

S4 Appendix cCENH3-ChIPped DNA FISH signal quantification on *Tribolium castaneum* chromosomes. DNA immunoprecipitated by using cCENH3 antibody (cCENH3-ChIPped DNA) was fluorochrome-labeled and *in situ* hybridized to *T*. *castaneum* chromosomes. cCENH3-ChIPped DNA signal proportion on *T*. *castaneum* metaphase chromosomes was calculated as a ratio of the cCENH3-ChIPped DNA signal length to the corresponding chromosome length. 122 chromosomes were analyzed.
(XLSX)

S5 Appendix Quantification analysis of chromatin fiber regions with overlapping cCENH3 and TCAST signals. Selected regions of 15 extended chromatin fibers with prominent cCENH3 and TCAST co-localization were quantified. Pixel intensities along the spline fitted lines were measured, and presence or absence of each signal was evaluated by extracting values that were higher or equal to 0, respectively. The degree of overlapping was calculated based on coincident presence of cCENH3 and TCAST signals.
(XLSX)

S6 Appendix Comparison of cCENH3-ChIP experiments using different Ab amounts and washing conditions. ChIP experiments were performed using different amounts of cCENH3 antibody (5 and 8 μg) and different washing conditions (weak wash with commercial washing buffers and strong wash with low/high salt buffers). The ChIP/Input ratio for the top 100 WGS clusters was compared between different ChIP experiments.
(XLSX)

S7 Appendix Comparison of ChIP-Seq Mapper outcome using different randomly sampled subsets of cCENH3-ChIP and Input reads. Following cCENH3-ChIP experiment and subsequent sequencing of ChIPped DNA fragments, ChIP-Seq Mapper analysis was performed using three different subsets of ChIP and Input randomly subsampled reads: 250000, 500000, and 1000000. ChIP and Input reads were mapped to the top 1000 WGS repeat clusters. Based on the ChIP/Input ratio, WGS clusters enriched for cCENH3 were determined.
(XLSX)
