# Supplementary figures and images for: CenH3 distribution reveals extended centromeres in the model beetle Tribolium castaneum
Source: PLoS Genet. 2020 Oct 30;16(10):e1009115. doi: 10.1371/journal.pgen.1009115 (PMC7598501; doi:10.1371/journal.pgen.1009115)

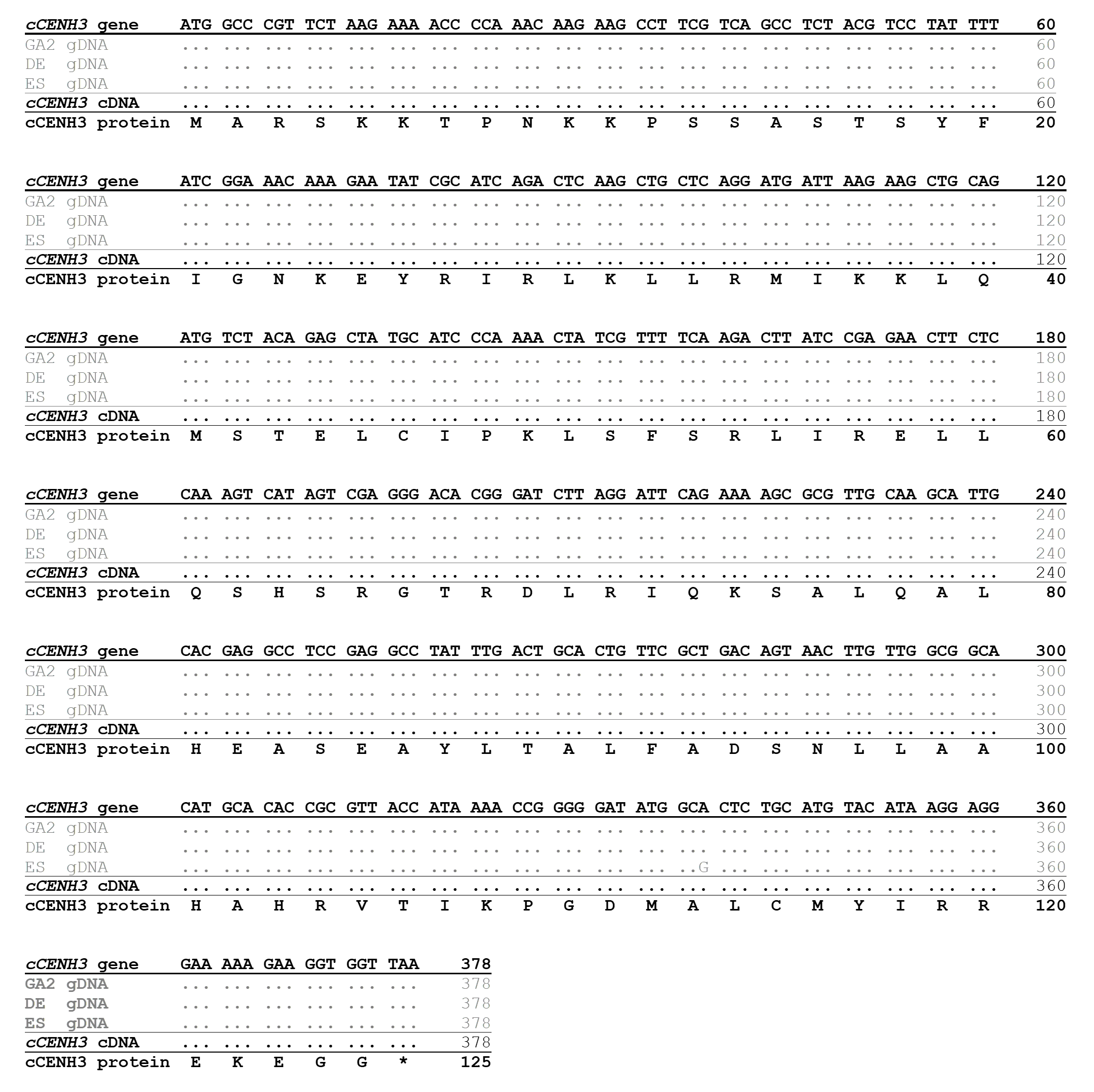

Supplement: S1 Fig — 378 bp long cCENH3 coding sequence (gDNA) was amplified from genomic DNAs isolated from three different T. castaneum strains (GA2, DE, and ES). Complementary DNA (cDNA) was synthesized from cCENH3 transcript by RT-PCR using primers specific for cCENH3 gene. Identical nucleotides are indicated by a dot, and only one synonymous substitution is present in ES strain. The cCENH3 protein translation is presented below the nucleotide alignment. (TIF) [file pgen.1009115.s001.tif]

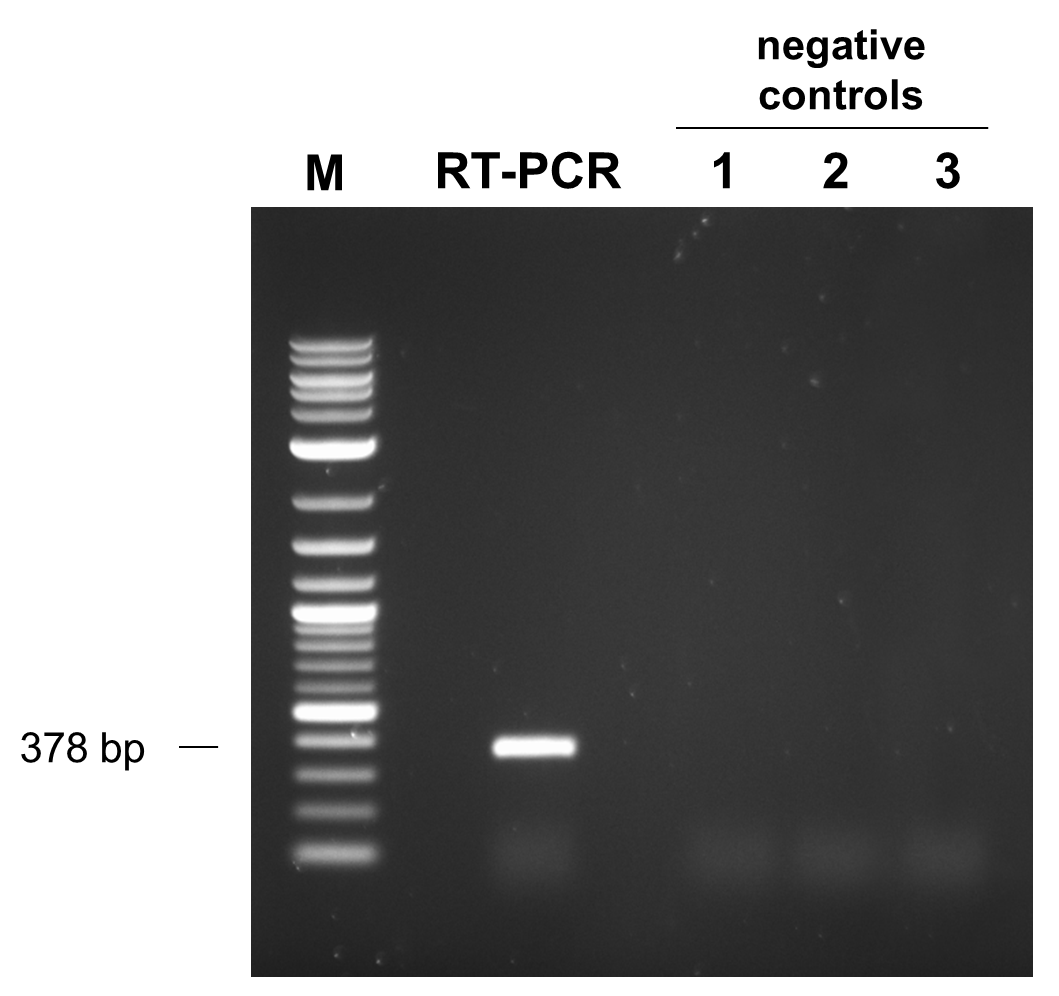

Supplement: S2 Fig — Reverse transcription PCR amplification was done on total RNA isolated from T. castaneum larvae by using the primers specific for the cCENH3 gene. The three negative controls included reactions: (1) without RT step, (2) with template RNA added after RT step, (3) RT-PCR without template RNA. M lane represents a 100-bp-size marker ladder. (TIF) [file pgen.1009115.s002.tif]

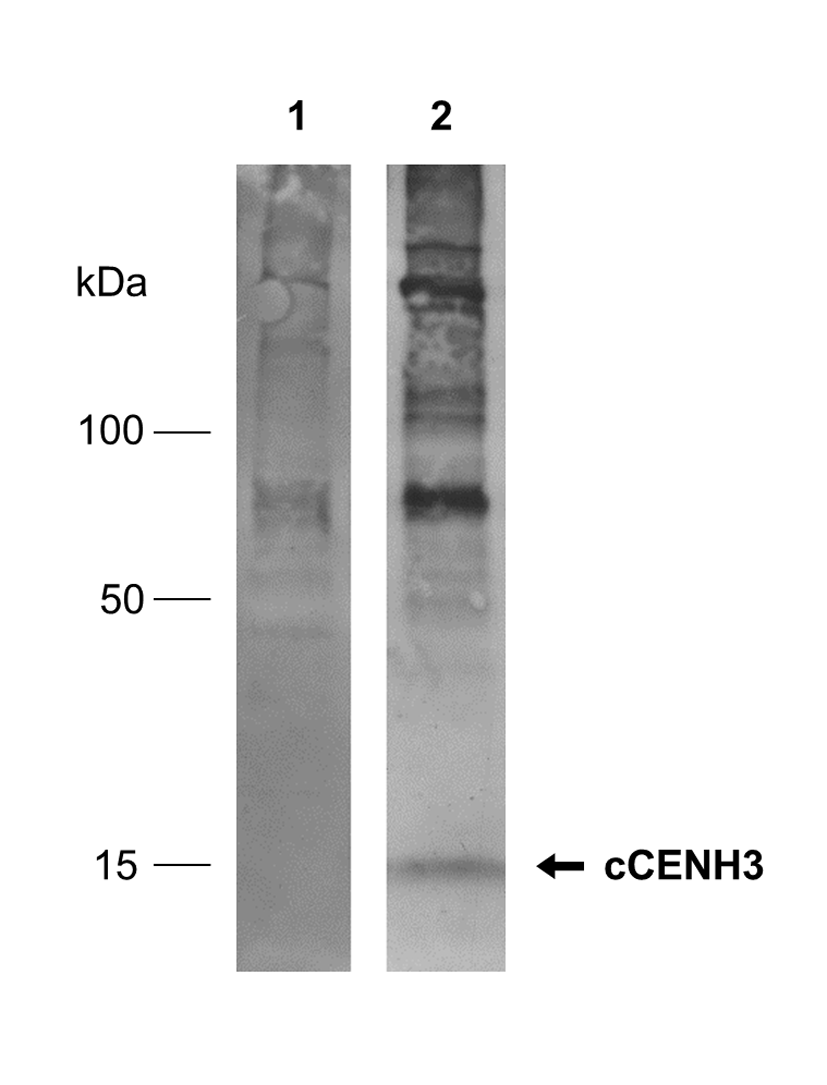

Supplement: S3 Fig — T. castaneum whole protein extract was fractionated by SDS-PAGE under denaturing conditions and transferred to nitrocellulose membrane. The membrane was subjected to Western blot analysis. (1) Testing of the rabbit preimmune serum. (2) Testing of the monospecific IgG fraction purified from the rabbit immunoserum after 120 days of immunization with the cCENH3-specific peptide (NH2-RSKKTPNKKPSSASTSYF-CONH2) revealed a ~15 kDa signal which is consistent with the expected molecular weight of cCENH3 protein. Molecular weights of protein sizes are indicated in kDa. (TIF) [file pgen.1009115.s003.tif]

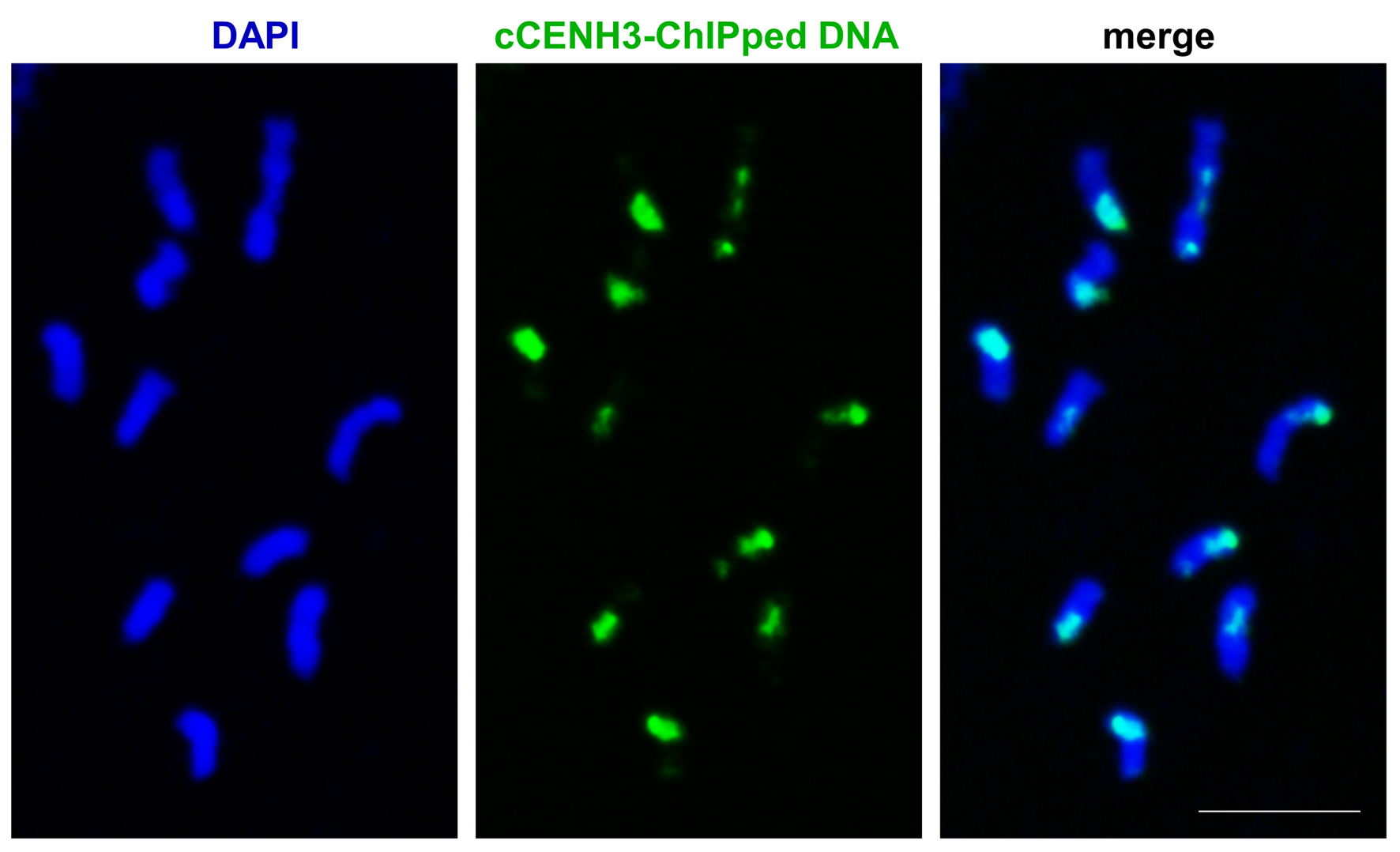

Supplement: S4 Fig — DNA immunoprecipitated by using cCENH3 antibody (cCENH3-ChIPped DNA) was Cy3-labelled and hybridized to T. castaneum chromosomes. cCENH3-ChIPped DNA (pseudocolored in green) hybridizes to the centromeric regions of all chromosomes (counterstained in DAPI). Scale bar = 5 μm. (TIF) [file pgen.1009115.s004.tif]

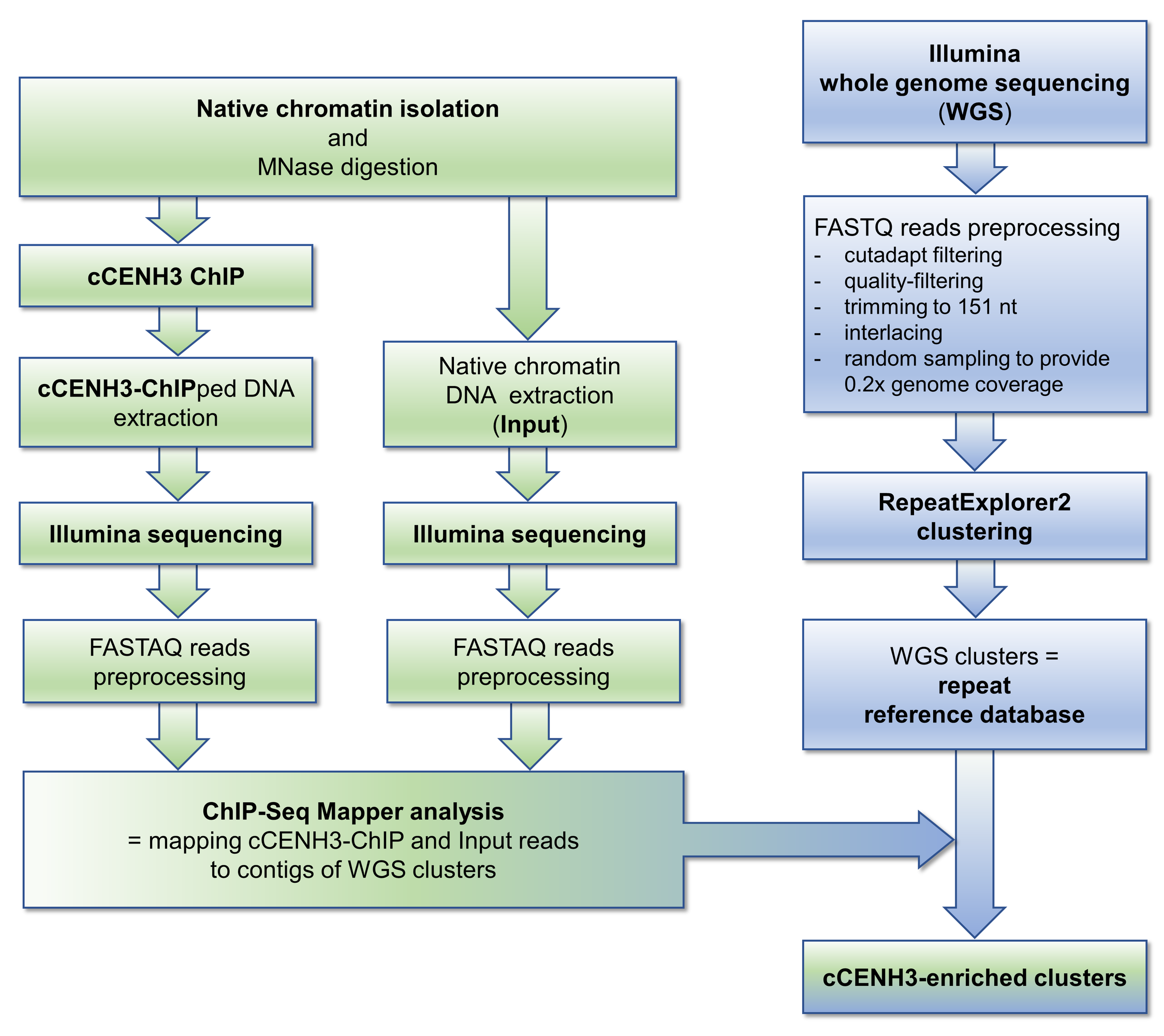

Supplement: S5 Fig — DNA sequences enriched for cCENH3 were identified following the strategy introduced by Neumann et al. [7]. First, the repetitive DNA reference database is formed by low-pass Illumina sequencing and RepeatExplorer2 similarity-based clustering of WGS unassembled reads. Chromatin immunoprecipitation (ChIP) was performed using the cCENH3 antibody. Immunoprecipitated DNA was Illumina sequenced, as well as DNA obtained from the chromatin preparation prior to ChIP (Input). ChIP and Input reads were mapped to the WGS clusters, and the cCENH3-enriched clusters were determined based on ChIP/Input reads elevated ratio. (TIF) [file pgen.1009115.s005.tif]

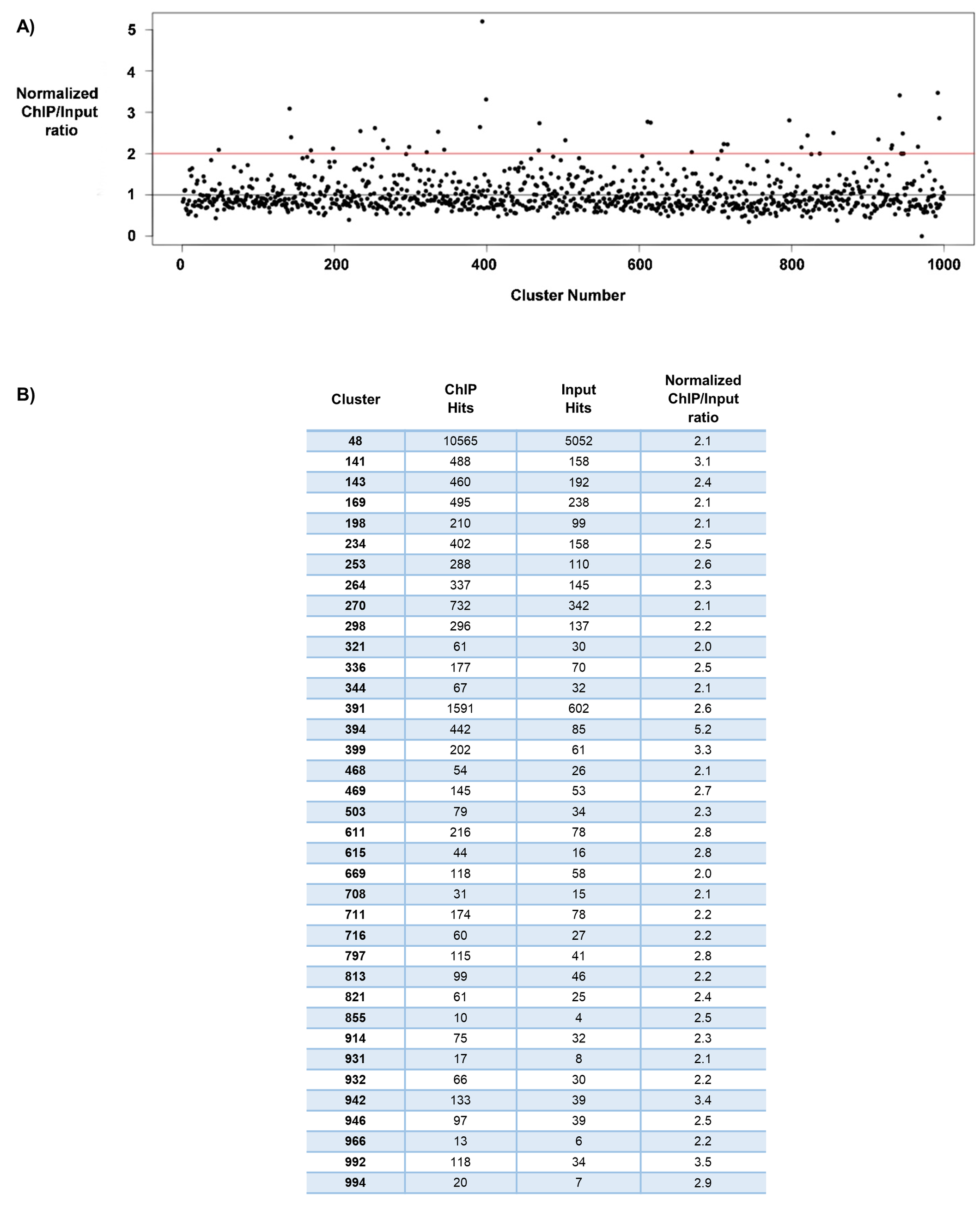

Supplement: S6 Fig — ChIP-Seq Mapper analysis based on one million cCENH3-ChIP and one million Input Illumina reads mapped to the top 1000 WGS T. castaneum repeat clusters obtained by RepeatExplorer2 analysis. (A) ChIP-Seq Mapper plot for the top 1000 WGS T. castaneum clusters analyzed for cCENH3 enrichment. The red line marks the mean ratio between ChIP and Input hits, and the clusters above red line show >2-fold enrichment for cCENH3. (B) List of 37 out of the top 1000 T. castaneum WGS clusters showing cCENH3-ChIP/Input ratio >2. (TIF) [file pgen.1009115.s006.tif]
