# Supplementary material for: CenH3 distribution reveals extended centromeres in the model beetle Tribolium castaneum
Source: PLoS Genet. 2020 Oct 30;16(10):e1009115. doi: 10.1371/journal.pgen.1009115 (PMC7598501; doi:10.1371/journal.pgen.1009115)
Supplement: S1 Table — (PDF) [file pgen.1009115.s007.pdf]

S1 Table. Estimation of *Tribolium castaneum* centromere sizes

Chromosome	Chromosome size [Mbp][1]	Number of measured chromosomes	Centromere proportion [%][2*]	Estimated centromere size [Mbp]*
**all**	204	240	43.47 (±8.68)	88.68 (±17.71)
**ch2**	22.42	15	42.89 (±6.97)	9.62 (±1.56)
**ch3**	37.41	31	42.34 (±7.92)	15.84 (±2.96)
**ch4**	22.52	22	46.48 (±5.99)	10.47 (±1.35)
**y_p**	5.71	8	40.60 (±8.09)	2.31 (±0.46)

1 The chromosome sizes were calculated from the relative length of the individual chromosomes [30] and haploid genome size of 204 Mb [23].

2 Centromere proportions were estimated from the ratio of the cCENH3 signal length demarcated by the outermost cCENH3-containing regions to the chromosome length. The quantifications are presented in the S3 Appendix.

* Standard deviations are indicated in brackets.
