# Supplementary material for: CenH3 distribution reveals extended centromeres in the model beetle Tribolium castaneum
Source: PLoS Genet. 2020 Oct 30;16(10):e1009115. doi: 10.1371/journal.pgen.1009115 (PMC7598501; doi:10.1371/journal.pgen.1009115)
Supplement: S1 Appendix — Distribution of 13 BLAST hits on the H3 query sequence is shown by the graphic alignment. The sequences in red represent H3 matches, while the potential CenH3 candidate (TC012577-PA) is marked in magenta. The matches in black represent partial and less significant hits. The alignments between H3 and all 13 matches are listed below, including the alignment score and expected values, and percentage of query/subject identities and positives. (PDF) [file pgen.1009115.s008.pdf]

S1 Appendix. BLASTP search against the *Tribolium castaneum* OGS3 database using histone H3 protein sequence as a query. Distribution of 13 Blast hits on the H3 query sequence is shown by the graphic alignment. The sequences in red represent H3 matches, while the potential CenH3 candidate (TC012577-PA) is marked in magenta. The matches in black represent partial and less significant hits. The alignments between H3 and all 13 matches are listed below, including the alignment score and expected values, and percentage of query/subject identities and positives.

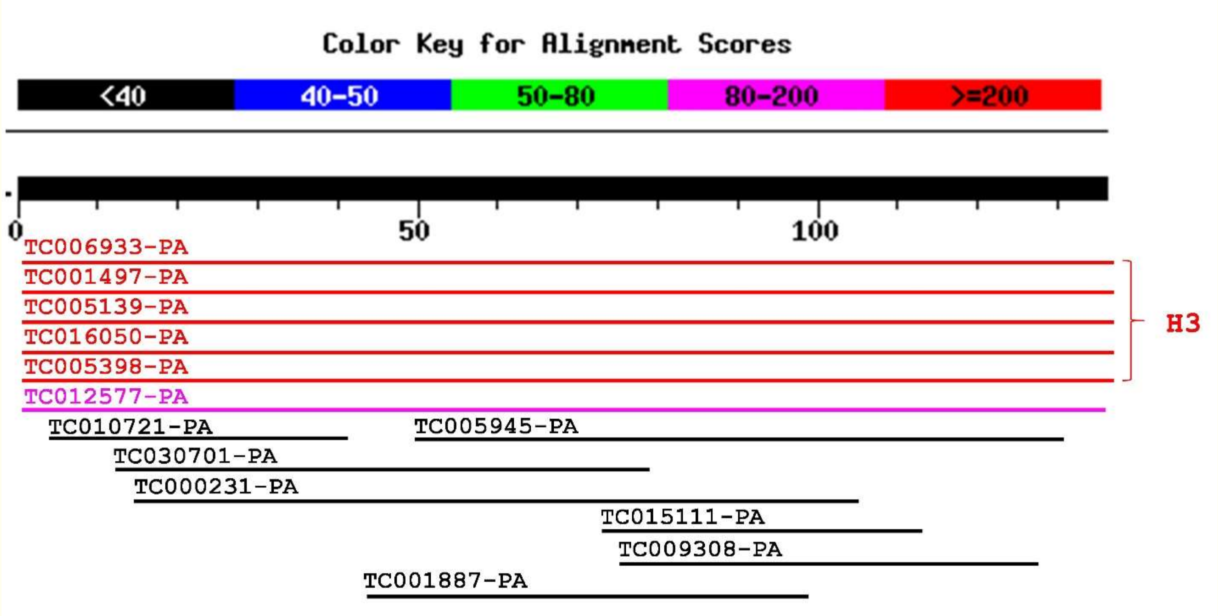

Sequences producing alignments:	Score (bits)	E Value
TC006933-PA (Unknown:14570956..14571366)	264	7e-72
TC001497-PA (Unknown:29373457..29373867)	264	7e-72
TC005139-PA (Unknown:23334197..23334607)	264	7e-72
TC016050-PA (Unknown:36741348..36741758)	264	7e-72
TC005398-PA (ChLG8:17239546..17240958)	258	4e-70
**TC012577-PA (ChLG9:16066895..16067272)**	**89**	**4e-19**
TC005945-PA (ChLG8:5643896..5650497)	28	0.80
TC030701-PA (ChLG3:35759566..35834262)	26	4.0
TC009308-PA (ChLG7:1817276..1819114)	25	6.7
TC015111-PA (ChLG6:5039228..5049650)	25	6.7
TC000231-PA (ChLG2:18781787..18783630)	25	6.7
TC010721-PA (Unknown:11584977..11586401)	25	8.8
TC001887-PA (Unknown:10737145..10747799)	25	8.8

```
>TC006933-PA (Unknown:14570956..14571366)
          Length = 136

 Score =  264 bits (675), Expect = 7e-72
 Identities = 136/136 (100%), Positives = 136/136 (100%)

[GBrowse][Subject FASTA]
Query: 1   MARTKQTARKSTGGKAPRKQLATKAARKSAPATGGVKKPHRYRPGTVALREIRRYQKSTE 60
           MARTKQTARKSTGGKAPRKQLATKAARKSAPATGGVKKPHRYRPGTVALREIRRYQKSTE
Sbjct: 1   MARTKQTARKSTGGKAPRKQLATKAARKSAPATGGVKKPHRYRPGTVALREIRRYQKSTE 60
Query: 61  LLIRKLPFQRLVREIAQDFKTDLRFQSSAVMALQEASEAYLVGLFEDTNLCAIHAKRVTI 120
           LLIRKLPFQRLVREIAQDFKTDLRFQSSAVMALQEASEAYLVGLFEDTNLCAIHAKRVTI
Sbjct: 61  LLIRKLPFQRLVREIAQDFKTDLRFQSSAVMALQEASEAYLVGLFEDTNLCAIHAKRVTI 120
Query: 121 MPKDIQLARRIRGERA 136
           MPKDIQLARRIRGERA
Sbjct: 121 MPKDIQLARRIRGERA 136



>TC001497-PA (Unknown:29373457..29373867)
          Length = 136

 Score =  264 bits (675), Expect = 7e-72
 Identities = 136/136 (100%), Positives = 136/136 (100%)

[GBrowse][Subject FASTA]
Query: 1   MARTKQTARKSTGGKAPRKQLATKAARKSAPATGGVKKPHRYRPGTVALREIRRYQKSTE 60
           MARTKQTARKSTGGKAPRKQLATKAARKSAPATGGVKKPHRYRPGTVALREIRRYQKSTE
Sbjct: 1   MARTKQTARKSTGGKAPRKQLATKAARKSAPATGGVKKPHRYRPGTVALREIRRYQKSTE 60
Query: 61  LLIRKLPFQRLVREIAQDFKTDLRFQSSAVMALQEASEAYLVGLFEDTNLCAIHAKRVTI 120
           LLIRKLPFQRLVREIAQDFKTDLRFQSSAVMALQEASEAYLVGLFEDTNLCAIHAKRVTI
Sbjct: 61  LLIRKLPFQRLVREIAQDFKTDLRFQSSAVMALQEASEAYLVGLFEDTNLCAIHAKRVTI 120
Query: 121 MPKDIQLARRIRGERA 136
           MPKDIQLARRIRGERA
Sbjct: 121 MPKDIQLARRIRGERA 136



>TC005139-PA (Unknown:23334197..23334607)
          Length = 136

 Score =  264 bits (675), Expect = 7e-72
 Identities = 136/136 (100%), Positives = 136/136 (100%)

[GBrowse][Subject FASTA]
Query: 1   MARTKQTARKSTGGKAPRKQLATKAARKSAPATGGVKKPHRYRPGTVALREIRRYQKSTE 60
           MARTKQTARKSTGGKAPRKQLATKAARKSAPATGGVKKPHRYRPGTVALREIRRYQKSTE
Sbjct: 1   MARTKQTARKSTGGKAPRKQLATKAARKSAPATGGVKKPHRYRPGTVALREIRRYQKSTE 60
Query: 61  LLIRKLPFQRLVREIAQDFKTDLRFQSSAVMALQEASEAYLVGLFEDTNLCAIHAKRVTI 120
           LLIRKLPFQRLVREIAQDFKTDLRFQSSAVMALQEASEAYLVGLFEDTNLCAIHAKRVTI
Sbjct: 61  LLIRKLPFQRLVREIAQDFKTDLRFQSSAVMALQEASEAYLVGLFEDTNLCAIHAKRVTI 120
Query: 121 MPKDIQLARRIRGERA 136
           MPKDIQLARRIRGERA
Sbjct: 121 MPKDIQLARRIRGERA 136



>TC016050-PA (Unknown:36741348..36741758)
          Length = 136

 Score =  264 bits (675), Expect = 7e-72
 Identities = 136/136 (100%), Positives = 136/136 (100%)
```

```
[GBrowse][Subject FASTA]
Query: 1   MARTKQTARKSTGGKAPRKQLATKAARKSAPATGGVKKPHRYRPGTVALREIRRYQKSTE 60
           MARTKQTARKSTGGKAPRKQLATKAARKSAPATGGVKKPHRYRPGTVALREIRRYQKSTE
Sbjct: 1   MARTKQTARKSTGGKAPRKQLATKAARKSAPATGGVKKPHRYRPGTVALREIRRYQKSTE 60
Query: 61  LLIRKLPFQRLVREIAQDFKTDLRFQSSAVMALQEASEAYLVGLFEDTNLCAIHAKRVTI 120
           LLIRKLPFQRLVREIAQDFKTDLRFQSSAVMALQEASEAYLVGLFEDTNLCAIHAKRVTI
Sbjct: 61  LLIRKLPFQRLVREIAQDFKTDLRFQSSAVMALQEASEAYLVGLFEDTNLCAIHAKRVTI 120
Query: 121 MPKDIQLARRIRGERA 136
           MPKDIQLARRIRGERA
Sbjct: 121 MPKDIQLARRIRGERA 136
```

>**TC005398-PA (ChLG8:17239546..17240958)**

```
          Length = 136

 Score =  258 bits (660), Expect = 4e-70
 Identities = 132/136 (97%), Positives = 135/136 (99%)

[GBrowse][Subject FASTA]
Query: 1   MARTKQTARKSTGGKAPRKQLATKAARKSAPATGGVKKPHRYRPGTVALREIRRYQKSTE 60
           MARTKQTARKSTGGKAPRKQLATKAARKSAP+TGGVKKPHRYRPGTVALREIRRYQKSTE
Sbjct: 1   MARTKQTARKSTGGKAPRKQLATKAARKSAPSTGGVKKPHRYRPGTVALREIRRYQKSTE 60
Query: 61  LLIRKLPFQRLVREIAQDFKTDLRFQSSAVMALQEASEAYLVGLFEDTNLCAIHAKRVTI 120
           LLIRKLPFQRLVREIAQDFKTDLRFQS+A+ ALQEASEAYLVGLFEDTNLCAIHAKRVTI
Sbjct: 61  LLIRKLPFQRLVREIAQDFKTDLRFQSAAIGALQEASEAYLVGLFEDTNLCAIHAKRVTI 120
Query: 121 MPKDIQLARRIRGERA 136
           MPKDIQLARRIRGERA
Sbjct: 121 MPKDIQLARRIRGERA 136
```

>**TC012577-PA (ChLG9:16066895..16067272)**

```
          Length = 125

 Score = 89.4 bits (220), Expect = 4e-19
 Identities = 59/138 (42%), Positives = 71/138 (51%), Gaps = 19/138 (13%)

[GBrowse][Subject FASTA]
Query: 1   MARTKQTARKSTGGKAPRKQLATKAARKSAPATGGVKKPHRYRPGTVALREIRRYQKSTE 60
           MAR+K+T  K     +    +  K                 YR     LR I++ Q STE
Sbjct: 1   MARSKKTPNKKPSSASTSYFIGNK----------------EYRIRLKLLRMIKKLQMSTE 44
Query: 61  LLIRKLPFQRLVREIAQDFKT---DLRFQSSAVMALQEASEAYLVGLFEDTNLCAIHAKR 117
           L I KL F RL+RE+ Q       DLR Q SA+ AL EASEAYL  LF D+NL A HA R
Sbjct: 45  LCIPKLSFSRLIRELLQSHSRGTRDLRIQKSALQALHEASEAYLTALFADSNLLAAHAHR 104
Query: 118 VTIMPKDIQLARRIRGER 135
           VTI P D+ L   IR E+
Sbjct: 105 VTIKPGDMALCMYIRREK 122
```

>**TC005945-PA (ChLG8:5643896..5650497)**

```
          Length = 769

 Score = 28.5 bits (62), Expect = 0.80
 Identities = 23/90 (25%), Positives = 44/90 (48%), Gaps = 14/90 (15%)

[GBrowse][Subject FASTA]
Query: 51  EIRRYQKSTELLIRKLPFQRLVREIAQDFKTDL--------RFQSSAVMALQEASEAYLV 102
           E + Y K+ E+L  K+PF+R+ + + QD    +        RF     + +Q  +  + +
Sbjct: 210 EEKNYVKALEVL--KIPFKRIPKRVTQDLVNMMLELLLLTDRFTECLDIFIQHCNFTFEI 267
Query: 103 GLFEDTNLCAIHAKRVTIMPKDIQLARRIR 132
            + ED N  AI +    +MP  IQ+  +++
```

```
Sbjct: 268 TVNED-NTIAIDS---YVMPPSIQIDLKVK 293
```

>**TC030701-PA (ChLG3:35759566..35834262)**
Length = 18024

Score = 26.2 bits (56), Expect = 4.0
Identities = 16/68 (23%), Positives = 32/68 (47%)

[GBrowse][Subject FASTA]

```
Query: 14    GKAPRKQLATKAARKSAPATGGVKKPHRYRPGTVALREIRRYQKSTELLIRKLPFQRLVR 73
             GK P+K    + A K   A   VK+       T  ++EI   ++  ++   K  F++  +
Sbjct: 15978 GKVPKKPEEEEEAVKLKKAPERVKEEELPEAKTPKVKEIEEKEEPEKIEFEKPEFEKPEK 16037
Query: 74    EIAQDFKT 81
             E+ ++ K+
Sbjct: 16038 EVPEEEKS 16045
```

>**TC009308-PA (ChLG7:1817276..1819114)**
Length = 518

Score = 25.4 bits (54), Expect = 6.7
Identities = 19/55 (34%), Positives = 27/55 (49%), Gaps = 1/55 (1%)

[GBrowse][Subject FASTA]

```
Query: 82  DLRFQSSAVMALQEASEAYLVGLFEDTNLCAIHAKRVTIMPKDIQLA-RRIRGER 135
           DLR +   V  LQ+  E+  V L ++  L    +K +     DIQL  +RIR  R
Sbjct: 326 DLRQKDQLVKHLQQEIESLKVELMKEKCLSENKSKELLEKSDDIQLLNKRIRSSR 380
```

>**TC015111-PA (ChLG6:5039228..5049650)**
Length = 509

Score = 25.4 bits (54), Expect = 6.7
Identities = 13/42 (30%), Positives = 21/42 (50%)

[GBrowse][Subject FASTA]

```
Query: 71  LVREIAQDFKTDLRFQSSAVMALQEASEAYLVGLFEDTNLCA 112
           +VR+   D    +   S  V   QE +EA++  L+  T +CA
Sbjct: 223 IVRQDGLDLPIGIYRPSIVVSTYQEPTEAWINNLYGPTGVCA 264
```

>**TC000231-PA (ChLG2:18781787..18783630)**
Length = 424

Score = 25.4 bits (54), Expect = 6.7
Identities = 22/92 (23%), Positives = 37/92 (40%), Gaps = 5/92 (5%)

[GBrowse][Subject FASTA]

```
Query: 15  KAPRKQLATKAARKSAPATGGVKKPHRYRPGTVALREIRRYQKSTELLIRKLPFQRLVRE 74
           + P+   A      S  A G   KP   RP  V L+ + +Y     +  ++LP+   V
Sbjct: 43  RQPKASTAHMVKSFSRSAAGKQIKPETLRPPQVLLKTV-KYLLCDVINTKRLPYWHSV-- 99
Query: 75  IAQDFKTDLRFQSSAVMALQEASEAYLVGLFE 106
              DF TD        + +Q  S+A  + + +
Sbjct: 100 --YDFITDRLLAVRQDLVVQNVSKAESITILQ 129
```

>**TC010721-PA (Unknown:11584977..11586401)**

```
          Length = 451

 Score = 25.0 bits (53), Expect = 8.8
 Identities = 12/38 (31%), Positives = 18/38 (47%)

[GBrowse][Subject FASTA]
Query: 3   RTKQTARKSTGGKAPRKQLATKAARKSAPATGGVKKPH 40
            RT  T+ ++T  K P +Q A   +  + P T     PH
Sbjct: 389 RTPLTSPQTTSLKIPSRQTAISPSTPATPTTSRTPFPH 426
```

>TC001887-PA (Unknown:10737145..10747799)

```
          Length = 1264

 Score = 25.0 bits (53), Expect = 8.8
 Identities = 12/57 (21%), Positives = 30/57 (52%)

[GBrowse][Subject FASTA]
Query: 42  YRPGTVALREIRRYQKSTELLIRKLPFQRLVREIAQDFKTDLRFQSSAVMALQEASE 98
            Y   +  L+E++R      + + +     L+R++A++ +  + F+  AV  + E++E
Sbjct: 56  YGKMSTRLKELQRKYHDENIKVARKDGLLLIRDMAKEVQNMMSFKIEAVRRITESAE 112



  Database: Official_Gene_Proteins.fa
    Posted date:  Jun 15, 2011  2:12 PM
  Number of letters in database: 7,426,099
  Number of sequences in database:  16,531

Lambda     K      H
   0.321    0.131    0.361

Gapped
Lambda     K      H
   0.267   0.0410    0.140


Matrix: BLOSUM62
Gap Penalties: Existence: 11, Extension: 1
Number of Hits to DB: 1,669,841
Number of Sequences: 16531
Number of extensions: 57156
Number of successful extensions: 177
Number of sequences better than 10.0: 13
Number of HSP's better than 10.0 without gapping: 10
Number of HSP's successfully gapped in prelim test: 3
Number of HSP's that attempted gapping in prelim test: 162
Number of HSP's gapped (non-prelim): 17
length of query: 136
length of database: 7,426,099
effective HSP length: 86
effective length of query: 50
effective length of database: 6,004,433
effective search space: 300221650
effective search space used: 300221650
T: 11
A: 40
X1: 16 ( 7.4 bits)
X2: 38 (14.6 bits)
X3: 64 (24.7 bits)
S1: 41 (21.9 bits)
S2: 53 (25.0 bits)
```
